# Long-term moderately elevated LDL-cholesterol and blood pressure and risk of coronary heart disease

**DOI:** 10.1371/journal.pone.0200017

**Published:** 2018-07-30

**Authors:** Peter Ueda, Pablo Gulayin, Goodarz Danaei

**Affiliations:** 1 Department of Global Health and Population, Harvard T.H. Chan School of Public Health, Boston, Massachusetts, United States of America; 2 Clinical Epidemiology Unit, Department of Medicine, Solna, Karolinska Institutet, Stockholm, Sweden; 3 Department of Global Health Policy, Graduate School of Medicine, The University of Tokyo, Tokyo, Japan; 4 Institute for Clinical Effectiveness and Health Policy (IECS) in Buenos Aires, Buenos Aires, Argentina; 5 Department of Epidemiology, Harvard T.H. Chan School of Public Health, Boston, Massachusetts, United States of America; The University of Tokyo, JAPAN

## Abstract

**Background:**

Harmful effects of long-term exposure to moderately elevated low-density lipoprotein (LDL)-cholesterol and blood pressure on coronary heart disease (CHD) have not been rigorously examined. We estimated the risk of CHD under long-term exposure to moderately elevated LDL-cholesterol and blood pressure compared with the risk under shorter exposures to higher levels of the same risk factors.

**Methods:**

Observational study using data from 2,714 adults in Framingham Offspring Study who were free of existing cardiovascular disease and aged <70 years at baseline (1987–1991). We used the parametric g-formula to estimate 16-year CHD risk under different levels and durations of exposure to LDL-cholesterol (low: <130 mg/dL, moderate: 130 to <160 mg/dL, high 160 to <190 mg/dL, and very high: ≥190 mg/dL) and systolic blood pressure (low: <120 mmHg, prehypertension: 120 to <140 mmHg, stage 1 hypertension: 140 to <160 mmHg, and stage 2 hypertension: ≥160 mmHg).

**Results:**

The estimated 16-year CHD risk under exposure to low LDL was 8.2% (95% CI = 7.0–9.6). The 16-year CHD risk under exposure to moderate LDL was 8.9% (7.8–10.1) which was similar to CHD risk under 8 years of low LDL followed by 8 years of high LDL at 9.0% (7.7–10.3); and 12 years of low LDL followed by 4 years of very high LDL at 8.8% (7.6–10.1). The results for blood pressure were similar.

**Conclusions:**

Long-term exposure to moderate levels of LDL-cholesterol and blood pressure had a similar impact on CHD risk as shorter exposures to levels considered ‘high’ per clinical guidelines.

## Introduction

Low-density lipoprotein (LDL) cholesterol and blood pressure are well-established causal risk factors for coronary heart disease (CHD).[1,2] Treatment recommendations for these risk factors are based on levels of exposure at which the risk increase and treatment benefit are considered large enough to warrant intervention. For example, an LDL-cholesterol level of 190 mg/dL or higher is considered an indication for lifestyle intervention or statin therapy in both the American and European guidelines.[3,4] Similarly, hypertension is defined as a blood pressure of 140/90 mmHg, and is staged based on thresholds to indicate the urgency for and type of intervention.[4]

The relationship between LDL-cholesterol and blood pressure with CHD is continuous and the risk increases at levels much lower than the thresholds used in clinical guidelines[1,2]; patients with moderately elevated LDL-cholesterol or blood pressure levels are therefore at increased CHD risk compared to those with low levels of these risk factors, and the elevated risk is expected to increase with prolonged exposure. Nonetheless, the magnitude of the long-term effect of moderately elevated risk factors on CHD is not clear. Previous studies that have assessed this relationship[5–8] used standard regression methods (e.g. Cox proportional hazard model) that may provide biased estimates as they cannot appropriately adjust for time-varying confounding affected by previous exposures.[9] For example, a high recording of LDL-cholesterol (previous exposure) may lead to changes in alcohol intake and smoking which are also a strong confounder for the effect of future LDL-cholesterol levels because they may affect both LDL-cholesterol and also CHD risk through other biological pathways. In contrast, the family of g-methods including the parametric g-formula have been specifically developed to adjust for time-varying confounding in longitudinal studies. The parametric g-formula has previously been used to estimate the effect of long-term changes in risk factors on the risk of diabetes,[10] CHD,[11] and asthma[12] as well as for evaluating treatment regimens for HIV-infection.[13]

In this study, we quantified the impact of long-term exposure to moderately elevated LDL-cholesterol and blood pressure on risk of CHD using the parametric g-formula and data on repeated risk factor measurements from 16 years of follow-up in the Framingham Offspring Study.

## Methods

### Study population

The Framingham Offspring Study is a prospective cohort study including 5,013 individuals recruited between 1971 and 1975, with follow-up examination cycles conducted almost every four years. More details on this cohort have been provided elsewhere.[14,15] Data from the Framingham Offspring Study were obtained from the National Heart Lung and Blood Institute (NHLBI) Biolincc database, and re-analyzed as part of the Biolincc data sharing policies.

We used the fourth examination cycle (in 1987–1991) as baseline to have complete pre-baseline data on important covariates and included information on time-varying covariates from baseline and three subsequent examinations. We included information on smoking, alcohol intake, body mass index (BMI), diabetes, systolic blood pressure (SBP), LDL-cholesterol, and blood pressure and lipid-lowering medications. LDL-cholesterol was calculated using the Friedewald equation and fasting serum samples. For non-fasting samples and triglyceride levels of more than 400 mg/dL, LDL-cholesterol was set to missing and the value from the previous examination cycle was carried forward. We also used age, sex, marital status and education at baseline, as well as smoking history and covariate data from the third examination cycle (pre-baseline). If information on one or more covariates was missing for a participant, we carried the last observed value forward for one examination cycle; if data for a covariate was missing for two consecutive examination cycles, we censored the participant after carrying the data over for one cycle.

**Fig 1:**
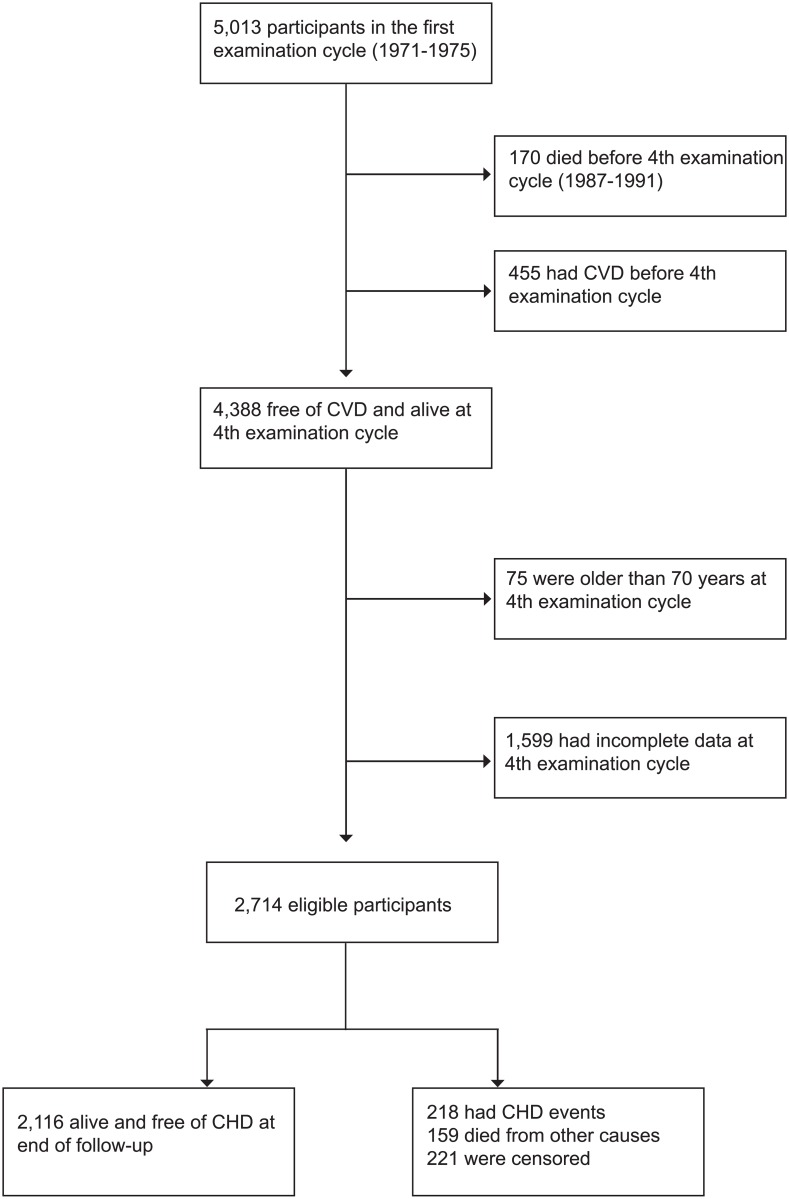
Flowchart of participant selection.

We included 4,388 participants who were alive and free of cardiovascular disease (defined as myocardial infarction, angina, coronary insufficiency, transient ischemic attack, stroke, or heart failure) at baseline. From these, 1,599 had incomplete covariate data after carrying data one cycle forward as explained above and 75 were 70 years old or older at baseline and were therefore excluded (Fig 1).

Participants were followed until the first CHD event, defined as myocardial infarction, coronary insufficiency, angina and CHD death as ascertained by the adjudication committee,[15] death, loss to follow-up, or administrative end of follow-up four years after the date of the 7^th^ examination, whichever occurred first.

### Statistical analysis

We used the parametric g-formula to estimate the cumulative risk of CHD under different scenarios for LDL and SBP. The parametric g-formula is an extension of standardization to time-varying settings and assuming that all time-varying confounders have been correctly measured and modelled appropriately, it provides an unbiased estimate of the risk of outcomes under different hypothetical interventions on time-varying risk factors.

The steps for estimating the parametric g-formula have previously been described,[10,11] and can be summarized as follows. First, we fit regression models for all time-varying covariates, death and CHD using pooled person-time data. Next, we use the coefficients from these models to simulate the risk of CHD under each of the hypothetical interventions using the following steps: (1) use the observed values of covariates at baseline; (2) estimate the joint distribution of the time-varying covariates at the next examination using parametric models; (3) ‘intervene‘ by setting the values of covariates to values determined by the hypothetical interventions; (4) estimate the predicted probability of death and CHD using these new values; (5) repeat steps 2 through 4 for the entire study period. To estimate the predicted risk of CHD under a different intervention, steps 1–4 should be repeated.

The definitions and functional forms of the covariates that we used in the regression models are presented in S1 Table. We used separate models for simulations of interventions on LDL-cholesterol and SBP. We did not include lipid-lowering medication in the LDL analyses or BP lowering medication in SBP analyses as we were aiming to estimate the impact of all major determinants of change in LDL and SBP including medications. To examine the validity of our parametric models, we compared the observed means of the time-varying confounders, risk of death and CHD with those predicted by the models.

We estimated the 16-year population risk of CHD under hypothetical interventions in which we altered the level and duration of exposure to LDL-cholesterol and SBP separately. We used the following levels of exposure to LDL-cholesterol: <130 mg/dL (low), 130 to <160 mg/dL (moderate), 160 to 189 mg/dL (high), and ≥190 mg/dL (very high);[3,16] and SBP exposure was categorized as: <120 mmHg (low); prehypertension (120 to 139 mmHg); stage 1 hypertension (140 to 159 mmHg); and stage 2 hypertension (160 mmHg or higher).[17,18] We used 4, 8, 12 and 16 years as the duration of exposure to each of the above levels of exposure which corresponds to the examination cycles in the cohort.

The hypothetical interventions were specified such that if a participant had a LDL or SBP value below the lower limit of the range specified by the intervention at any examination, the value was set the lower limit, and similarly for values that were above the upper limit.

In another set of analyses, we examined whether changing the age at which participants are exposed to higher levels matters by moving the periods of higher exposure to the beginning of follow-up. For each intervention, we also calculated the proportion of the study participants who had their risk factor value intervened on at any examination cycle and the average proportion of participants who had their risk factor intervened on in each examination cycle. These analyses were done to measure to what extent participants in the study followed the risk factor trajectories specified by our interventions.

We used non-parametric bootstrapping with 1000 samples to estimate the 95% confidence intervals. We used SAS 9.4 (Cary, NC) for all the analyses. The SAS macro and its documentation are available from the Harvard TH Chan School of Public Health (www.hsph.harvard.edu/causal/software).[19] The study was approved by the Institutional Review Board at the Harvard TH Chan School of Public Health, and participants have provided written consent to the Framingham Heart Study.

## Results

Table 1 shows the baseline characteristics of the 2,714 eligible participants and covariates from each examination cycle. Mean (SD) age was 50.7 (9.5) years at baseline and 53% were women. Median LDL-cholesterol levels decreased by 12 mg/dL during the study period and the proportion of the participants that used lipid-lowering medications increased from 3% to 19%. Despite an increase in proportion of participants taking antihypertensive medications (from 15% to 33%), median SBP levels remained unchanged at around 125 mmHg. During 16 years of follow-up (39,884 person-years), 221 participants were lost to follow-up; 218 had a CHD event and 159 died from other causes.

**Table 1:** Characteristics of eligible 2,714 participants in the Framingham Offspring Study. Values are represented in percentage unless otherwise indicated.

Characteristics	3^rd^ exam (pre-baseline; 1984–1987)	4^th^ exam (baseline; 1987–1991)	5^th^ exam (1991–1994)	6^th^ exam (1994–1998)	7^th^ exam (1998–2001)
Current smoker	27.5	23.0	19.1	14.9	12.2
Cigarettes per day among smokers, mean (SD)	22.9 (12.9)	21.9 (12.6)	21.1 (12.0)	19.9 (11.7)	18.3 (11.3)
Alcoholic drinks per day					
None	27.6	29.8	30.2	36.8	33.7
>0 to <2	54.2	55.5	55.3	53.2	52.9
2 to <4	13.9	11.3	11.7	8.6	10.8
4 or more	4.3	3.4	2.9	1.4	2.6
Body mass index (kg/m^2^); mean (SD)	26 (4.6)	26.6 (4.8)	27.3 (4.9)	27.8 (5.2)	28.1 (5.3)
Diabetes	2.7	3.9	6.3	9.6	12.3
Systolic blood pressure (mmHg); median (IQR)	121 (111, 132)	124 (112, 137)	124 (112.5, 137)	126 (114, 138.5)	125 (114, 138)
Systolic blood pressure categories					
<120 mmHg (Low)	45.8	40.5	40.2	34.3	34.1
120 to <140 mmHg (Pre-hypertension)	40.6	38.6	38.6	39.3	37.5
140 to <160 mmHg (Stage 1 hypertension)	11.0	16.1	16.4	16.2	15.5
≥160 mmHg (Stage 2 hypertension)	2.6	4.9	4.9	6.0	5.2
LDL-cholesterol (mg/dL); median (IQR)	133 (110.2, 158.6)	130.6 (107, 156.2)	125.4 (104.4, 148.6)	125 (104.8, 147.8)	119.2 (98.4, 141)
LDL-cholesterol categories					
<130 mg/dL (low)	46.3	49.2	53.7	52.7	56.6
130 to <160 mg/dL (moderate)	29.7	29.8	29.8	26.8	22.4
160 to <190 mg/dL (high)	16.8	15.6	11.5	11.3	8.2
≥190 mg/dL (very high)	7.2	5.4	3.8	3.6	2.1
Blood pressure medication	13.5	15.1	16.3	25.6	32.6
Lipid-lowering medication	0.7	2.6	5.7	10.8	18.7

The models estimated mean risk factor levels under no intervention that were close to those observed in the data indicating lack of substantial model misspecification. For example, mean difference between observed and simulated LDL-cholesterol was <0.7 mg/dl during follow-up. The corresponding difference for mean systolic blood pressure level was <0.8 mmHg (S1 and S2 Figs). The simulated 16-year risk of CHD was 8.8% for the model used for LDL-cholesterol and 8.7% for the SBP model, which are both quite close to the observed risk of 8.54%. The coefficients of the models are presented in S2 and S3 Tables.

The estimated 16-year CHD risk for exposure to low LDL-levels (<130 mg/dL) was 8.2% (95% CI = 7.0–9.6) (Table 2). The 16-year CHD risk under exposure to moderate LDL (130 to <160 mg/dL) for the entire follow-up was 8.9% (7.8–10.1) which was similar to CHD risk under 8 years of low LDL followed by 8 years of high LDL (160 to <190mg/dL) at 9.0% (7.7–10.3); and 12 years of low LDL followed by 4 years of very high LDL (≥190 mg/dL) at 8.8% (7.6–10.1).

**Table 2:** Sixteen-year risk of coronary heart disease (CHD) under different levels and durations of exposure to LDL-cholesterol, Framingham Offspring Study (1987–1991 to 2007).

LDL level and duration*	16-year risk of CHD^a^	Population risk ratio^b^	Population risk difference^b^	Cumulative percentage intervened on	Average percentage intervened on
Low LDL (<130 mg/dL) for 16 yrs^c^	8.2 (7.0 to 9.6)	1	0	73	38
Low LDL for 12 yrs followed very high LDL (>190 mg/dL) for 4 yrs	8.8 (7.6 to 10.1)	1.07 (0.95 to 1.25)	0.6 (-0.4 to 1.9)	100	56
Moderate LDL (130 to <160 mg/dL) for 16 yrs	8.9 (7.8 to 10.1)	1.08 (0.95 to 1.22)	0.7 (-0.4 to 1.7)	98	64
Low LDL for 8 yrs followed high LDL (160 to <190 mg/dL) for 8 yrs	9.0 (7.7 to 10.3)	1.09 (0.95 to 1.27)	0.7 (-0.5 to 2.0)	100	66
Low LDL for 8 yrs followed by very high LDL for 8 yrs	9.4 (7.8 to 11.4)	1.14 (0.92 to 1.47)	1.2 (-0.7 to 3.5)	100	70
High LDL for 16 yrs	9.4 (7.8 to 11.5)	1.14 (0.92 to 1.46)	1.2 (-0.7 to 3.5)	99	79
Very high LDL for 16 yrs	10.3 (7.6 to 13.9)	1.25 (0.87 to 1.80)	2.0 (-1.1 to 6.0)	99	79

* Sorted by ascending CHD risk

^a^. There were 218 cases of CHD among 2,972 cohort participants after 39,884 person-years of follow-up. The observed risk was 8.5%.

^b^. In addition to LDL-cholesterol levels, we modeled 7 other covariates in the analysis: examination cycle, cigarette smoking (current smoker, and number of cigarettes per day if smoker), alcohol consumption (standard drinks per day), body mass index, diabetes, systolic blood pressure, and blood pressure medication. All models included lagged values of time-varying covariates plus baseline non-time-varying variables: sex, age, education level, marital status at examination cycle 4, and smoking history at examination cycle 3 of the Framingham Offspring Study.

^c^. Reference category

The estimated 16-year CHD risk for exposure to low SBP (<120 mmHg) during the entire follow-up was 6.7% (95% CI = 5.5–8.1) (Table 3). The 16-year CHD risk under exposure to prehypertension (120 to <140 mmHg) for the entire follow-up was 8.4% (7.3–9.5) which was almost equal to CHD risk under 8 years of low SBP followed by 8 years of stage 1 hypertension (140 to <160 mmHg), or CHD risk under 12 years of low SBP followed by 4 years of stage 2 hypertension (≥160 mmHg), which were both 8.6%. The results for all combinations of timing, level and duration of LDL are reported in S5 Table. The estimated 16-year CHD risks did not change materially when periods of increased exposure to LDL or SBP were moved to the beginning of the follow-up instead of the end (S4 and S5 Tables).

**Table 3:** Sixteen-year risk of coronary heart disease (CHD) under different levels and durations of exposure to systolic blood pressure (SBP), Framingham Offspring Study (1987–1991 to 2007).

SBP level and duration*	16-year risk of CHD^a^	Population risk ratio^b^	Population risk difference^b^	Cumulative percentage intervened on	Average percentage intervened on
Low SBP for 16 yrs (<120 mmHg)^c^	6.7 (5.5 to 8.1)	1	0	85	55
Prehypertension (120 to <140 mmHg) for 16 yrs	8.4 (7.3 to 9.5)	1.26 (1.11 to 1.41)	1.7 (0.9 to 2.5)	95	55
Low SBP for 8 yrs followed by stage 1 hypertension (140 to <160 mmHg) for 8 yrs	8.6 (7.5 to 9.7)	1.28 (1.12 to 1.49)	1.9 (0.9 to 2.9)	100	68
Low SBP for 12 yrs followed by stage 2 hypertension (≥160 mmHg) for 4 yrs	8.6 (7.5 to 10.0)	1.29 (1.11 to 1.57)	1.9 (0.8 to 3.3)	100	67
Stage 1 hypertension (140 to <160 mmHg) for 16 yrs	10.4 (8.9 to 12.1)	1.55 (1.23 to 1.95)	3.7 (1.8 to 5.6)	98	73
Low SBP for 8 yrs followed stage 2 hypertension (≥160 mmHg) for 8 yrs	10.5 (8.7 to 12.5)	1.57 (1.21 to 2.03)	3.8 (1.7 to 6.1)	100	75
Stage 2 hypertension (≥160 mmHg) for 16 yrs	13.7 (10.1 to 17.0)	2.04 (1.40 to 2.90)	7.0 (3.1 to 10.9)	98	73

* Sorted by ascending CHD risk

^a^. There were 218 cases of CHD among 2,972 cohort participants after 39,884 person-years of follow-up. The observed risk was 8.5%.

^b^. In addition to systolic blood pressure levels, we modeled 7 other covariates in the analysis: examination cycle, cigarette smoking (current smoker, and number of cigarettes per day if smoker), alcohol consumption (standard drinks per day), body mass index, diabetes, systolic blood pressure, and lipid lowering medication. All models included lagged values of time-varying covariates plus baseline non-time-varying variables: sex, age, education level, marital status at examination 4, and smoking history at examination 3 of the Framingham Offspring Study.

^c^. Reference category

## Discussion

We found that long-term exposure to moderate levels of LDL-cholesterol and SBP has the same impact on CHD risk as shorter exposures to levels considered ‘high’ per clinical guidelines, suggesting that individuals exposed to moderate levels of the risk factors during a longer period may also benefit from intensive lifestyle modification or medication. For example, 16 years of exposure to moderate LDL-levels produced a similar 16-year CHD risk as 8 years of high LDL (preceded by 8 years of low LDL) or 4 years of very high LDL (preceded by 12 years of low LDL).

We estimated a relative CHD risk of 1.25 for 16-years of exposure to LDL-cholesterol>190 mg/dL compared with 16 years of LDL-cholesterol<130 mg/dL which is lower than the hazard ratio of 2.27 for 60 mg/dL increase in ‘usual’ LDL-cholesterol calculated based on results of a large meta-analysis of observational studies.[20] Similarly, our estimated relative risk of 2.04 comparing 16 years of exposure to stage 2 hypertension with low SBP (i.e. approximately 40 mmHg difference in mean SBP) is much lower than a hazard ratio of 3.13 for CHD reported for the same mean ‘usual’ SBP difference in a pooling study of prospective cohorts for ages 65–74 years.[21] However, the effects estimated in our study have a different interpretation as the g-formula adjusts for baseline levels of LDL-cholesterol and SBP and therefore estimates the effect of a *change* as opposed to conventional analyses of prospective studies that estimate the effect of differences in the levels of risk factors across individuals. The latter estimates reflect the lifelong effect of a risk factor and is expectedly larger than effect of change at baseline where for example in our study participants are already 50 years old.

The validity of our estimates depends on the assumptions of no unmeasured confounding, no measurement error, and no model misspecification. Our models estimated risk factor levels and risks of CHD under no intervention that were close to those observed in the data, which is a requirement for no model misspecification. Our study has several limitations. First, because the number of CHD events was too small to allow for meaningful subgroup analyses, we did not examine the effect of exposure to risk factors for specific age groups. Such analyses would be relevant because the age at which LDL-cholesterol levels start to increase may affect its long-term effects.[22] Second, although we included several major potential confounders in our analyses, we did not use information on diet and physical activity as it is available for all examination cycles in the Framingham Offspring Study; this may have led to unmeasured confounding. Similarly, we could not include other disease outcomes such as stroke or congestive heart failure or cancer as the number of events were too small. Third, we defined our ‘intervention’ based on risk factor levels without specifying how participants achieved those levels. Therefore, our estimates should be interpreted as the effects of a combination of changes that the study participants indeed experienced that led to the observed LDL-cholesterol and blood pressure changes, including lifestyle modification and medication use. Fourth, under all scenarios (with the exception of 16 years of low LDL-cholesterol or low SBP), almost all participants had their LDL-cholesterol or SBP value ‘intervened on’ in at least one examination cycle. Nonetheless, a substantial proportion of the study population (21–62% for LDL-cholesterol model and 27–45% for SBP) did follow the intervention in at least one examination cycle (i.e. the compliment of the percentage reported in the last column of Tables 2 and 3). Fifth, we did not have information from enough time points to estimate the CHD risk reduction from lowering LDL-cholesterol and blood pressure following prolonged exposure to moderately elevated levels of the risk factors. Finally, we had to start our analytic sample at the fourth examination cycle to adjust for pre-baseline values of risk factors. This limited our population sample to younger participants and those who did not have a fatal event in the first four cycles of the study, which limits the generalizability of our findings to similar populations. We note however, that such restriction, which is required to adjust for time-varying confounding, is unlikely to introduce selection bias as our models adjusted for most common causes of cardiovascular disease at baseline.

Our findings have important implications for future CHD prevention guidelines. Physicians and patients should consider duration of exposure in addition to the level of LDL-cholesterol and SBP when evaluating the potential benefits of lifestyle modification and medication use. Individuals with moderate levels of blood pressure and LDL-cholesterol that have persisted for years or decades may require as intensive treatment as those with high or very high levels of risk factors. Pooling studies of randomized clinical trials have shown that statin therapy reduces risk of cardiovascular disease across levels of disease risk at treatment initiation, although the risk reduction increases with pre-treatment LDL-cholesterol levels.[23] In the HOPE-3 trial, which was specifically designed to assess the effects of cholesterol and blood pressure treatment in individuals at intermediate cardiovascular disease risk, statin therapy in participants with low levels of pre-treatment LDL-cholesterol significantly reduced the risk of cardiovascular disease,[24] but lowering blood pressure in individuals with pre-hypertension at baseline did not reduce cardiovascular disease risk.[25] In contrast, the SPRINT trial found that lowering SBP to less than 120 mmHg reduced the risk of cardiovascular disease compared with a target of <140 mmHg in individuals with different levels of baseline blood pressure, including those with baseline measurements in the pre-hypertensive range.[26] It is worth noting that the cardiovascular risk scores used in these trials do not include the duration of exposure as a risk predictor and may therefore underestimate the risk in individuals with long-term exposure to moderate levels of risk factors.

Preventing cardiovascular disease in individuals with long-term exposure to moderately elevated risk factors will be even more important in the next few decades because levels of major cardiovascular risk factors including cholesterol and blood pressure are elevated already in adolescents in many countries.[27–29] Intensive lifestyle modification is quite challenging for most individuals and it can only be expected that it is even more challenging for patients with long-term exposure to moderately elevated risk factor levels. In addition, poor adherence remains a challenge in treating healthy individuals with elevated cardiovascular disease risk[30,31] and strategies and tools to improve adherence (e.g. by improving communication of cardiovascular risk and potential treatment benefits[32]) are needed.

## Supporting information

S1 Table Covariates used to model incidence of coronary heart disease in the Framingham Offspring Study during 16 years of follow-up after the 4th examination cycle (1987–1991). (DOCX)

S2 Table Coefficients of regressions used in the simulations on LDL-cholesterol. (DOCX)

S3 Table Coefficients of regressions used in the simulations on systolic blood pressure. (DOCX)

S4 Table Risk of coronary heart disease (CHD) under different levels, durations and timing of exposure to LDL-cholesterol in the Framingham Offspring Study during 16 years of follow-up time after the 4th examination cycle (1987–1991). (DOCX)

S5 Table Risk of coronary heart disease (CHD) under different levels, durations and timing of exposure to systolic blood pressure (SBP) in the Framingham Offspring Study during 16 years of follow-up time after the 4th examination cycle (1987–1991). (DOCX)

S1 Fig Mean difference between observed and simulated values and their 95% confidence intervals by examination cycle for the models used for interventions on LDL-cholesterol in the Framingham Offspring Study: (a) number of cigarettes smoked per day, (b) number of alcoholic drinks per day, (c) body mass index (BMI), (d) prevalence of diabetes mellitus, (e) systolic blood pressure, (f) LDL-cholesterol and (g) blood pressure medication. Observed values were used for the 4th examination cycle (baseline), and follow-up included examination cycles 5–7.
(DOCX)

S2 Fig Mean difference between observed and simulated values and their 95% confidence intervals by examination cycle for the models used for interventions on systolic blood pressure in the Framingham Offspring Study: (a) number of cigarettes smoked per day, (b) number of alcoholic drinks per day, (c) body mass index (BMI), (d) prevalence of diabetes mellitus, (e) systolic blood pressure, (f) LDL-cholesterol and (g) lipid lowering medication. Observed values were used for the 4th examination cycle (baseline) and follow-up included examination cycles 5–7.
(DOCX)
