# Supplementary material for: Long-term moderately elevated LDL-cholesterol and blood pressure and risk of coronary heart disease
Source: PLoS One. 2018 Jul 30;13(7):e0200017. doi: 10.1371/journal.pone.0200017 (PMC6066205; doi:10.1371/journal.pone.0200017)
Supplement: S1 Table — (DOCX) [file pone.0200017.s001.docx]

**S1 Table. Covariates used to model incidence of coronary heart disease in the Framingham Offspring Study during 16 years of follow-up after the 4th examination cycle (1987-1991).**

| **Variable name (code name)** | **Examination cycles assessed^a^** | **As dependent** | **As independent** |
| --- | --- | --- | --- |
| **Fixed covariates** |  |  |  |
| Sex | 4th | Not predicted | 2 categories |
| Baseline age (age_bl, ageage_bl^b^) | 4th | Not predicted | Continuous and quadratic |
| Education (edu) | 4th | Not predicted | 4 categories^c^ |
| Marital status (mastat) | 4th | Not predicted | 3 categories^d^ |
| Ever smoked before baseline (eversmok) | 3rd | Not predicted | 2 categories |
| Prebaseline alcoholic drinks per day (drinksday_prebl) | 3rd | Not predicted | 4 categories^e^ |
| Prebaseline body mass index (bmi_prebl) | 3rd | Not predicted | Continuous |
| Prebaseline diabetes mellitus (dm_prebl) | 3rd | Not predicted | 2 categories |
| Prebaseline systolic blood pressure (sbp_prebl) | 3rd | Not predicted | Continuous |
| Prebaseline cigarretes per day (cigday_prebl) | 3rd | Not predicted | 5 categories^f^ |
| Prebaseline LDL-cholesterol (ldlf_prebl) | 3rd | Not predicted | Continuous |
| Prebaseline blood pressure medication (bpmed_prebl) | 3rd | Not predicted | 2 categories |
| Prebaseline anti-cholesterol medication (antichol_prebl) | 3rd | Not predicted | 2 categories |
|  | | | |
| **Time varying covariates** |  |  |  |
| Examination cycle (exam) | ------ | Not predicted | 4 categories^g^ |
| Cigarretes per day (cigday) | 4th, 5th, 6th, 7th | Logistic then log linear^h^ | 5 categories |
| Alcoholic drinks per day (drinksday) | 4th, 5th, 6th, 7th | Logistic then log linear^h^ | 4 categories |
| Body mass index (bmi) | 4th, 5th, 6th, 7th | Linear regression | Continuous |
| Diabetes mellitus (dm) | 4th, 5th, 6th, 7th | Logistic to failure^i^ |  |
| Systolic blood pressure (sbp) | 4th, 5th, 6th, 7th | Linear regression | Continuous |
| LDL-cholesterol (ldlf) | 4th, 5th, 6th, 7th | Linear regression | Continuous |
| Blood pressure medication^j^ (bpmed) | 4th, 5th, 6th, 7th | Logistic regression | 2 categories |
| Lipid lowering medication^k^ (antichol) | 4th, 5th, 6th, 7th | Logistic regression | 2 categories |

^a^ The examination cycles took place during the following years: 3rd (1984-1987); 4th (1987-1991): 5th (1991-1994); 6th (1995-1998); and 7th (1998-2001).

^b^ Squared age variable

^c^ Categories were: master´s degree/ doctorate, bachelor´s degree, junior college/high school diploma, none of above.

^d^ Categories were: single, married and widowed/divorced/separated.

^e^ Categories were: none, 1 to <2, 2 to <4, and 4 or more per day.

^f^ Categories were: non-smokers, <1, 1 to <5, 5 to <24 and 25 or more cigarettes /day.

^g^ Categories were: the 4th, 5th, 6th, and 7th examination cycle.

^h^ These covariates were predicted using two stages, first a logistic regression on an indicator of whether the variable is nonzero and then a linear regression of the log of the nonzero values.

^i^ For the diabetes variable, the value was predicted based on a logistic regression only if the predicted probability for the prior period was 0.

^j^ Included only in the models used for interventions on LDL-cholesterol.

^k^ Included only in the models used for interventions on systolic blood pressure.
