# Supplementary material for: Long-term moderately elevated LDL-cholesterol and blood pressure and risk of coronary heart disease
Source: PLoS One. 2018 Jul 30;13(7):e0200017. doi: 10.1371/journal.pone.0200017 (PMC6066205; doi:10.1371/journal.pone.0200017)
Supplement: S2 Table — (DOCX) [file pone.0200017.s002.docx]

**S2 Table Coefficients of regressions used in the simulations on LDL-cholesterol** ^a,b,c^

^a^ For the list of code names, see S1 Table. For variables with more than two categories, the highest category is used as the reference.

^b^ The suffixes in the variable names are as follows: the numerical suffixes represent the categories of the values of the covariate as defined in the footnotes of S1 Table; l1 indicates the value lagged for one examination cycle

^c^ *tsdm_l1_inter* is the product of the lagged value of *dm* (diabetes) variable and the cumulative sum of the *dm* variable from the baseline to the preceding examination cycle.

1. Logistic model to estimate the probability of coronary heart disease

| **Parameter** | **Estimate** | **Standard Error** | **Wald Chi-Square** | **Pr > ChiSq** |
| --- | --- | --- | --- | --- |
| **Intercept** | -10.3612 | 2.7085 | 14.6340 | 0.0001 |
| **sex** | -0.9929 | 0.1642 | 36.5577 | <.0001 |
| **age_bl** | 0.1737 | 0.0976 | 3.1672 | 0.0751 |
| **ageage_bl** | -0.00137 | 0.000909 | 2.2733 | 0.1316 |
| **edu1** | 0.2990 | 0.3280 | 0.8309 | 0.3620 |
| **edu2** | 0.1600 | 0.2461 | 0.4226 | 0.5157 |
| **edu3** | 0.3192 | 0.2728 | 1.3694 | 0.2419 |
| **mastat1** | -0.5446 | 0.3959 | 1.8917 | 0.1690 |
| **mastat2** | -0.4287 | 0.2039 | 4.4178 | 0.0356 |
| **eversmok** | 0.1131 | 0.1694 | 0.4451 | 0.5046 |
| **cigday_prebl1** | 0.0830 | 0.6024 | 0.0190 | 0.8904 |
| **cigday_prebl2** | -0.3586 | 0.4329 | 0.6860 | 0.4075 |
| **cigday_prebl3** | -0.1286 | 0.2727 | 0.2223 | 0.6373 |
| **drinksday_prebl1** | 0.0508 | 0.3707 | 0.0188 | 0.8910 |
| **drinksday_prebl2** | 0.0129 | 0.3414 | 0.0014 | 0.9699 |
| **drinksday_prebl3** | 0.2811 | 0.3391 | 0.6874 | 0.4071 |
| **bmi_prebl** | 0.1366 | 0.0313 | 19.0548 | <.0001 |
| **dm_prebl** | 0.2699 | 0.3419 | 0.6230 | 0.4299 |
| **sbp_prebl** | -0.00950 | 0.00582 | 2.6635 | 0.1027 |
| **ldlf_prebl** | 0.00680 | 0.00238 | 8.1668 | 0.0043 |
| **bpmed_prebl** | 0.0320 | 0.2001 | 0.0255 | 0.8731 |
| **antichol_prebl** | 0.9180 | 0.5439 | 2.8493 | 0.0914 |
| **exam_1** | -0.4670 | 0.2237 | 4.3592 | 0.0368 |
| **exam_2** | -0.2665 | 0.2115 | 1.5871 | 0.2077 |
| **exam_3** | -0.1379 | 0.1977 | 0.4862 | 0.4856 |
| **cigday** | 0.0208 | 0.00680 | 9.3924 | 0.0022 |
| **drinksday_1** | -0.1341 | 0.4034 | 0.1104 | 0.7397 |
| **drinksday_2** | -0.3259 | 0.3798 | 0.7362 | 0.3909 |
| **drinksday_3** | -0.6491 | 0.4044 | 2.5765 | 0.1085 |
| **bmi** | -0.1097 | 0.0299 | 13.5029 | 0.0002 |
| **dm** | 0.4143 | 0.3915 | 1.1200 | 0.2899 |
| **tsdm_inter** | 0.0561 | 0.1638 | 0.1175 | 0.7318 |
| **sbp** | 0.0169 | 0.00438 | 14.8585 | 0.0001 |
| **ldlf** | 0.00264 | 0.00254 | 1.0777 | 0.2992 |
| **bpmed** | 0.6394 | 0.1870 | 11.6889 | 0.0006 |

1. Logistic model to estimate the probability of death from a cause other than coronary heart disease

| **Parameter** | **Estimate** | **Standard Error** | **Wald Chi-Square** | **Pr > ChiSq** |
| --- | --- | --- | --- | --- |
| **Intercept** | -7.5450 | 3.4298 | 4.8394 | 0.0278 |
| **sex** | -0.5833 | 0.1832 | 10.1395 | 0.0015 |
| **age_bl** | 0.1557 | 0.1223 | 1.6194 | 0.2032 |
| **ageage_bl** | -0.00064 | 0.00111 | 0.3376 | 0.5612 |
| **edu1** | 0.6209 | 0.3557 | 3.0464 | 0.0809 |
| **edu2** | 0.1992 | 0.2940 | 0.4591 | 0.4980 |
| **edu3** | -0.2772 | 0.3594 | 0.5948 | 0.4406 |
| **mastat1** | -0.4110 | 0.4676 | 0.7725 | 0.3794 |
| **mastat2** | -0.3489 | 0.2308 | 2.2849 | 0.1306 |
| **eversmok** | 0.3479 | 0.2061 | 2.8486 | 0.0915 |
| **cigday_prebl1** | -0.2096 | 0.7301 | 0.0824 | 0.7740 |
| **cigday_prebl2** | 0.0158 | 0.4354 | 0.0013 | 0.9710 |
| **cigday_prebl3** | 0.1624 | 0.2771 | 0.3436 | 0.5578 |
| **drinksday_prebl1** | -0.3153 | 0.4164 | 0.5735 | 0.4489 |
| **drinksday_prebl2** | -0.0706 | 0.3736 | 0.0357 | 0.8501 |
| **drinksday_prebl3** | -0.1199 | 0.3743 | 0.1027 | 0.7486 |
| **bmi_prebl** | 0.1772 | 0.0360 | 24.2062 | <.0001 |
| **dm_prebl** | 0.0805 | 0.4500 | 0.0320 | 0.8580 |
| **sbp_prebl** | -0.00049 | 0.00657 | 0.0056 | 0.9405 |
| **ldlf_prebl** | -0.00010 | 0.00291 | 0.0013 | 0.9716 |
| **bpmed_prebl** | 0.2828 | 0.2448 | 1.3342 | 0.2481 |
| **antichol_prebl** | -0.5134 | 1.0313 | 0.2478 | 0.6186 |
| **exam_1** | -1.1443 | 0.2573 | 19.7715 | <.0001 |
| **exam_2** | -0.6161 | 0.2254 | 7.4701 | 0.0063 |
| **exam_3** | -0.5949 | 0.2267 | 6.8878 | 0.0087 |
| **cigday** | 0.0198 | 0.00811 | 5.9815 | 0.0145 |
| **drinksday_1** | -0.3930 | 0.4531 | 0.7526 | 0.3857 |
| **drinksday_2** | -0.6607 | 0.4277 | 2.3869 | 0.1224 |
| **drinksday_3** | -0.5708 | 0.4391 | 1.6895 | 0.1937 |
| **bmi** | -0.1884 | 0.0351 | 28.7334 | <.0001 |
| **dm** | 0.7810 | 0.4904 | 2.5364 | 0.1112 |
| **tsdm_inter** | -0.2002 | 0.2125 | 0.8883 | 0.3459 |
| **sbp** | -0.00180 | 0.00540 | 0.1106 | 0.7395 |
| **ldlf** | -0.00394 | 0.00315 | 1.5633 | 0.2112 |
| **bpmed** | -0.0802 | 0.2315 | 0.1201 | 0.7289 |

1. Logistic model to estimate the probability of smoking

| **Parameter** | **Estimate** | **Standard Error** | **Wald Chi-Square** | **Pr > ChiSq** |
| --- | --- | --- | --- | --- |
| **Intercept** | -1.8607 | 1.5343 | 1.4707 | 0.2252 |
| **sex** | 0.3304 | 0.1233 | 7.1842 | 0.0074 |
| **age_bl** | -0.1228 | 0.0542 | 5.1345 | 0.0235 |
| **ageage_bl** | 0.000829 | 0.000550 | 2.2746 | 0.1315 |
| **edu1** | 0.6702 | 0.2723 | 6.0587 | 0.0138 |
| **edu2** | 0.2251 | 0.1959 | 1.3197 | 0.2507 |
| **edu3** | 0.00852 | 0.2223 | 0.0015 | 0.9694 |
| **mastat1** | 0.3091 | 0.2323 | 1.7698 | 0.1834 |
| **mastat2** | -0.1759 | 0.1546 | 1.2950 | 0.2551 |
| **eversmok** | 2.9677 | 0.4218 | 49.5018 | <.0001 |
| **cigday_prebl1** | 1.1948 | 0.2446 | 23.8577 | <.0001 |
| **cigday_prebl2** | 1.8528 | 0.1638 | 128.0084 | <.0001 |
| **cigday_prebl3** | 1.5714 | 0.1302 | 145.6359 | <.0001 |
| **drinksday_prebl1** | -0.0949 | 0.3341 | 0.0807 | 0.7763 |
| **drinksday_prebl2** | -0.1492 | 0.3099 | 0.2318 | 0.6302 |
| **drinksday_prebl3** | 0.0497 | 0.3104 | 0.0257 | 0.8727 |
| **bmi_prebl** | 0.0613 | 0.0273 | 5.0299 | 0.0249 |
| **dm_prebl** | -0.0832 | 0.7515 | 0.0123 | 0.9118 |
| **sbp_prebl** | 0.00296 | 0.00523 | 0.3199 | 0.5717 |
| **ldlf_prebl** | -0.00003 | 0.00223 | 0.0002 | 0.9876 |
| **bpmed_prebl** | 0.0840 | 0.2218 | 0.1433 | 0.7050 |
| **antichol_prebl** | 0.3186 | 0.6756 | 0.2224 | 0.6372 |
| **exam_1** | 0 | . | . | . |
| **exam_2** | 0.0842 | 0.1409 | 0.3573 | 0.5500 |
| **exam_3** | -0.1016 | 0.1407 | 0.5214 | 0.4702 |
| **cigday_l1** | 0.1680 | 0.00550 | 931.7794 | <.0001 |
| **drinksday_l1_1** | -0.5398 | 0.3665 | 2.1694 | 0.1408 |
| **drinksday_l1_2** | -0.5883 | 0.3474 | 2.8680 | 0.0904 |
| **drinksday_l1_3** | -0.3833 | 0.3568 | 1.1540 | 0.2827 |
| **bmi_l1** | -0.0791 | 0.0246 | 10.3059 | 0.0013 |
| **dm_l1** | -0.1041 | 0.6092 | 0.0292 | 0.8643 |
| **tsdm_l1_inter** | 0.1095 | 0.3605 | 0.0922 | 0.7615 |
| **sbp_l1** | -0.00200 | 0.00438 | 0.2081 | 0.6483 |
| **ldlf_l1** | 0.00134 | 0.00222 | 0.3622 | 0.5473 |
| **bpmed_l1** | -0.4165 | 0.2022 | 4.2420 | 0.0394 |

1. Log-linear model to estimate the number of cigarettes smoked per day among smokers

| **Variable** | **Parameter Estimate** | **Standard Error** | **t Value** | **Pr > \|t\|** |
| --- | --- | --- | --- | --- |
| **Intercept** | 1.39259 | 0.53076 | 2.62 | 0.0088 |
| **sex** | -0.03221 | 0.03939 | -0.82 | 0.4136 |
| **age_bl** | 0.01004 | 0.01761 | 0.57 | 0.5686 |
| **ageage_bl** | -0.00011369 | 0.00018163 | -0.63 | 0.5315 |
| **edu1** | 0.35760 | 0.09608 | 3.72 | 0.0002 |
| **edu2** | 0.29972 | 0.07307 | 4.10 | <.0001 |
| **edu3** | 0.21200 | 0.08328 | 2.55 | 0.0110 |
| **mastat1** | -0.05317 | 0.06975 | -0.76 | 0.4461 |
| **mastat2** | -0.02916 | 0.04773 | -0.61 | 0.5414 |
| **eversmok** | -0.13448 | 0.24464 | -0.55 | 0.5826 |
| **cigday_prebl1** | -0.95587 | 0.10872 | -8.79 | <.0001 |
| **cigday_prebl2** | -0.29783 | 0.06633 | -4.49 | <.0001 |
| **cigday_prebl3** | 0.03495 | 0.04127 | 0.85 | 0.3972 |
| **drinksday_prebl1** | 0.00882 | 0.09219 | 0.10 | 0.9238 |
| **drinksday_prebl2** | -0.03554 | 0.08306 | -0.43 | 0.6688 |
| **drinksday_prebl3** | 0.01999 | 0.08328 | 0.24 | 0.8104 |
| **bmi_prebl** | 0.01208 | 0.00885 | 1.36 | 0.1727 |
| **dm_prebl** | 0.19649 | 0.21166 | 0.93 | 0.3534 |
| **sbp_prebl** | 0.00067621 | 0.00165 | 0.41 | 0.6824 |
| **ldlf_prebl** | 0.00018268 | 0.00076316 | 0.24 | 0.8109 |
| **bpmed_prebl** | 0.09077 | 0.07740 | 1.17 | 0.2412 |
| **antichol_prebl** | -0.04757 | 0.26287 | -0.18 | 0.8564 |
| **exam_1** | 0 | . | . | . |
| **exam_2** | 0.05646 | 0.04646 | 1.22 | 0.2245 |
| **exam_3** | -0.00961 | 0.04802 | -0.20 | 0.8414 |
| **cigday_l1** | 0.03851 | 0.00165 | 23.32 | <.0001 |
| **drinksday_l1_1** | 0.00066109 | 0.09708 | 0.01 | 0.9946 |
| **drinksday_l1_2** | -0.08751 | 0.08970 | -0.98 | 0.3295 |
| **drinksday_l1_3** | -0.01106 | 0.09353 | -0.12 | 0.9059 |
| **bmi_l1** | -0.00606 | 0.00809 | -0.75 | 0.4538 |
| **dm_l1** | 0.05190 | 0.20691 | 0.25 | 0.8020 |
| **tsdm_l1_inter** | -0.05837 | 0.11453 | -0.51 | 0.6104 |
| **sbp_l1** | 0.00175 | 0.00145 | 1.20 | 0.2290 |
| **ldlf_l1** | -0.00064211 | 0.00071341 | -0.90 | 0.3683 |
| **bpmed_l1** | -0.18681 | 0.07301 | -2.56 | 0.0106 |

1. Logistic model to estimate the probability of starting to drinkg alcohol among those who did not drink

| **Parameter** | **Estimate** | **Standard Error** | **Wald Chi-Square** | **Pr > ChiSq** |
| --- | --- | --- | --- | --- |
| **Intercept** | 0.3910 | 1.4478 | 0.0729 | 0.7871 |
| **sex** | 0.1567 | 0.1192 | 1.7291 | 0.1885 |
| **age_bl** | -0.0337 | 0.0521 | 0.4186 | 0.5176 |
| **ageage_bl** | 0.000127 | 0.000518 | 0.0600 | 0.8064 |
| **edu1** | -0.6823 | 0.2788 | 5.9884 | 0.0144 |
| **edu2** | -0.5347 | 0.1787 | 8.9503 | 0.0028 |
| **edu3** | -0.0853 | 0.2003 | 0.1816 | 0.6700 |
| **mastat1** | 0.3736 | 0.2375 | 2.4750 | 0.1157 |
| **mastat2** | 0.3902 | 0.1693 | 5.3117 | 0.0212 |
| **eversmok** | 0.2171 | 0.1223 | 3.1488 | 0.0760 |
| **cigday_prebl1** | 0.6011 | 0.4440 | 1.8327 | 0.1758 |
| **cigday_prebl2** | 0.2014 | 0.2626 | 0.5879 | 0.4432 |
| **cigday_prebl3** | 0.2468 | 0.1899 | 1.6891 | 0.1937 |
| **drinksday_prebl1** | -1.0450 | 0.4848 | 4.6474 | 0.0311 |
| **drinksday_prebl2** | 0.1749 | 0.4832 | 0.1310 | 0.7174 |
| **drinksday_prebl3** | -0.2087 | 0.5554 | 0.1412 | 0.7071 |
| **bmi_prebl** | -0.0230 | 0.0248 | 0.8576 | 0.3544 |
| **dm_prebl** | -0.0256 | 0.6703 | 0.0015 | 0.9695 |
| **sbp_prebl** | -0.00779 | 0.00502 | 2.4050 | 0.1209 |
| **ldlf_prebl** | 0.00676 | 0.00208 | 10.5355 | 0.0012 |
| **bpmed_prebl** | -0.1170 | 0.1963 | 0.3554 | 0.5511 |
| **antichol_prebl** | 0.8660 | 0.5980 | 2.0974 | 0.1476 |
| **exam_1** | 0 | . | . | . |
| **exam_2** | 0.1707 | 0.1299 | 1.7279 | 0.1887 |
| **exam_3** | -0.5041 | 0.1373 | 13.4865 | 0.0002 |
| **cigday_l1** | -0.00345 | 0.0101 | 0.1171 | 0.7322 |
| **drinksday_l1_1** | 0 | . | . | . |
| **drinksday_l1_2** | 0 | . | . | . |
| **drinksday_l1_3** | 0 | . | . | . |
| **bmi_l1** | 0.00630 | 0.0225 | 0.0786 | 0.7792 |
| **dm_l1** | 0.1082 | 0.5602 | 0.0373 | 0.8468 |
| **tsdm_l1_inter** | -0.3604 | 0.3370 | 1.1438 | 0.2849 |
| **sbp_l1** | 0.00620 | 0.00414 | 2.2411 | 0.1344 |
| **ldlf_l1** | -0.00216 | 0.00219 | 0.9703 | 0.3246 |
| **bpmed_l1** | 0.1904 | 0.1769 | 1.1594 | 0.2816 |
| **cigday** | -0.00197 | 0.0113 | 0.0302 | 0.8620 |

1. Logistic model to estimate the probability of continuing drinking alcohol among those who drink

| **Parameter** | **Estimate** | **Standard Error** | **Wald Chi-Square** | **Pr > ChiSq** |
| --- | --- | --- | --- | --- |
| **Intercept** | 4.9667 | 1.2875 | 14.8805 | 0.0001 |
| **sex** | -0.3881 | 0.1022 | 14.4247 | 0.0001 |
| **age_bl** | 0.0145 | 0.0463 | 0.0978 | 0.7545 |
| **ageage_bl** | -0.00033 | 0.000454 | 0.5257 | 0.4684 |
| **edu1** | -0.3154 | 0.2336 | 1.8229 | 0.1770 |
| **edu2** | -0.3722 | 0.1515 | 6.0368 | 0.0140 |
| **edu3** | -0.1711 | 0.1716 | 0.9940 | 0.3188 |
| **mastat1** | -0.6012 | 0.2056 | 8.5505 | 0.0035 |
| **mastat2** | -0.00821 | 0.1407 | 0.0034 | 0.9535 |
| **eversmok** | 0.2671 | 0.1053 | 6.4312 | 0.0112 |
| **cigday_prebl1** | 0.0123 | 0.3280 | 0.0014 | 0.9702 |
| **cigday_prebl2** | 0.0129 | 0.2450 | 0.0028 | 0.9582 |
| **cigday_prebl3** | -0.3914 | 0.1560 | 6.2941 | 0.0121 |
| **drinksday_prebl1** | -1.6788 | 0.3017 | 30.9656 | <.0001 |
| **drinksday_prebl2** | -0.2471 | 0.2908 | 0.7224 | 0.3954 |
| **drinksday_prebl3** | 0.5285 | 0.3115 | 2.8773 | 0.0898 |
| **bmi_prebl** | -0.0137 | 0.0228 | 0.3603 | 0.5484 |
| **dm_prebl** | 0.6206 | 0.5050 | 1.5102 | 0.2191 |
| **sbp_prebl** | 0.000367 | 0.00417 | 0.0077 | 0.9300 |
| **ldlf_prebl** | 0.000978 | 0.00183 | 0.2850 | 0.5935 |
| **bpmed_prebl** | 0.2116 | 0.1690 | 1.5681 | 0.2105 |
| **antichol_prebl** | 0.3278 | 0.5772 | 0.3226 | 0.5701 |
| **exam_1** | 0 | . | . | . |
| **exam_2** | -0.2195 | 0.1294 | 2.8777 | 0.0898 |
| **exam_3** | -0.7128 | 0.1198 | 35.3908 | <.0001 |
| **cigday_l1** | -0.00708 | 0.00835 | 0.7189 | 0.3965 |
| **drinksday_l1_1** | 0 | . | . | . |
| **drinksday_l1_2** | 0.0516 | 0.3174 | 0.0265 | 0.8708 |
| **drinksday_l1_3** | 1.0221 | 0.3522 | 8.4224 | 0.0037 |
| **bmi_l1** | -0.0198 | 0.0204 | 0.9420 | 0.3318 |
| **dm_l1** | -0.0495 | 0.4426 | 0.0125 | 0.9109 |
| **tsdm_l1_inter** | -0.3151 | 0.2465 | 1.6339 | 0.2012 |
| **sbp_l1** | -0.00277 | 0.00340 | 0.6625 | 0.4157 |
| **ldlf_l1** | -0.00269 | 0.00189 | 2.0256 | 0.1547 |
| **bpmed_l1** | -0.3189 | 0.1525 | 4.3717 | 0.0365 |
| **cigday** | 0.000369 | 0.00943 | 0.0015 | 0.9688 |

1. Log-linear model to estimate the number of drinks per day among those drinking alcohol

| **Parameter** | **Estimate** | **Standard Error** | **Wald Chi-Square** | **Pr > ChiSq** |
| --- | --- | --- | --- | --- |
| **Intercept** | 4.9667 | 1.2875 | 14.8805 | 0.0001 |
| **sex** | -0.3881 | 0.1022 | 14.4247 | 0.0001 |
| **age_bl** | 0.0145 | 0.0463 | 0.0978 | 0.7545 |
| **ageage_bl** | -0.00033 | 0.000454 | 0.5257 | 0.4684 |
| **edu1** | -0.3154 | 0.2336 | 1.8229 | 0.1770 |
| **edu2** | -0.3722 | 0.1515 | 6.0368 | 0.0140 |
| **edu3** | -0.1711 | 0.1716 | 0.9940 | 0.3188 |
| **mastat1** | -0.6012 | 0.2056 | 8.5505 | 0.0035 |
| **mastat2** | -0.00821 | 0.1407 | 0.0034 | 0.9535 |
| **eversmok** | 0.2671 | 0.1053 | 6.4312 | 0.0112 |
| **cigday_prebl1** | 0.0123 | 0.3280 | 0.0014 | 0.9702 |
| **cigday_prebl2** | 0.0129 | 0.2450 | 0.0028 | 0.9582 |
| **cigday_prebl3** | -0.3914 | 0.1560 | 6.2941 | 0.0121 |
| **drinksday_prebl1** | -1.6788 | 0.3017 | 30.9656 | <.0001 |
| **drinksday_prebl2** | -0.2471 | 0.2908 | 0.7224 | 0.3954 |
| **drinksday_prebl3** | 0.5285 | 0.3115 | 2.8773 | 0.0898 |
| **bmi_prebl** | -0.0137 | 0.0228 | 0.3603 | 0.5484 |
| **dm_prebl** | 0.6206 | 0.5050 | 1.5102 | 0.2191 |
| **sbp_prebl** | 0.000367 | 0.00417 | 0.0077 | 0.9300 |
| **ldlf_prebl** | 0.000978 | 0.00183 | 0.2850 | 0.5935 |
| **bpmed_prebl** | 0.2116 | 0.1690 | 1.5681 | 0.2105 |
| **antichol_prebl** | 0.3278 | 0.5772 | 0.3226 | 0.5701 |
| **exam_1** | 0 | . | . | . |
| **exam_2** | -0.2195 | 0.1294 | 2.8777 | 0.0898 |
| **exam_3** | -0.7128 | 0.1198 | 35.3908 | <.0001 |
| **cigday_l1** | -0.00708 | 0.00835 | 0.7189 | 0.3965 |
| **drinksday_l1_1** | 0 | . | . | . |
| **drinksday_l1_2** | 0.0516 | 0.3174 | 0.0265 | 0.8708 |
| **drinksday_l1_3** | 1.0221 | 0.3522 | 8.4224 | 0.0037 |
| **bmi_l1** | -0.0198 | 0.0204 | 0.9420 | 0.3318 |
| **dm_l1** | -0.0495 | 0.4426 | 0.0125 | 0.9109 |
| **tsdm_l1_inter** | -0.3151 | 0.2465 | 1.6339 | 0.2012 |
| **sbp_l1** | -0.00277 | 0.00340 | 0.6625 | 0.4157 |
| **ldlf_l1** | -0.00269 | 0.00189 | 2.0256 | 0.1547 |
| **bpmed_l1** | -0.3189 | 0.1525 | 4.3717 | 0.0365 |
| **cigday** | 0.000369 | 0.00943 | 0.0015 | 0.9688 |

1. Linear model to estimate body mass index

| **Variable** | **Parameter Estimate** | **Standard Error** | **t Value** | **Pr > \|t\|** |
| --- | --- | --- | --- | --- |
| **Intercept** | 1.77872 | 0.62140 | 2.86 | 0.0042 |
| **sex** | 0.22675 | 0.04807 | 4.72 | <.0001 |
| **age_bl** | -0.01637 | 0.02212 | -0.74 | 0.4595 |
| **ageage_bl** | -0.00007534 | 0.00021852 | -0.34 | 0.7303 |
| **edu1** | 0.02127 | 0.10854 | 0.20 | 0.8447 |
| **edu2** | 0.02473 | 0.06810 | 0.36 | 0.7166 |
| **edu3** | 0.03500 | 0.07654 | 0.46 | 0.6475 |
| **mastat1** | 0.04120 | 0.10318 | 0.40 | 0.6897 |
| **mastat2** | -0.21062 | 0.06675 | -3.16 | 0.0016 |
| **eversmok** | 0.10408 | 0.05008 | 2.08 | 0.0377 |
| **cigday_prebl1** | -0.03431 | 0.15259 | -0.22 | 0.8221 |
| **cigday_prebl2** | 0.08654 | 0.11266 | 0.77 | 0.4424 |
| **cigday_prebl3** | 0.20277 | 0.07888 | 2.57 | 0.0102 |
| **drinksday_prebl1** | -0.09206 | 0.14110 | -0.65 | 0.5142 |
| **drinksday_prebl2** | -0.09079 | 0.13234 | -0.69 | 0.4927 |
| **drinksday_prebl3** | -0.20822 | 0.13391 | -1.55 | 0.1200 |
| **bmi_prebl** | 0.21020 | 0.01112 | 18.90 | <.0001 |
| **dm_prebl** | -0.30232 | 0.25416 | -1.19 | 0.2343 |
| **sbp_prebl** | -0.00321 | 0.00203 | -1.58 | 0.1132 |
| **ldlf_prebl** | 0.00166 | 0.00087463 | 1.89 | 0.0584 |
| **bpmed_prebl** | 0.05719 | 0.08235 | 0.69 | 0.4874 |
| **antichol_prebl** | 0.12312 | 0.28344 | 0.43 | 0.6640 |
| **exam_1** | 0 | . | . | . |
| **exam_2** | 0.17420 | 0.05630 | 3.09 | 0.0020 |
| **exam_3** | 0.18838 | 0.05545 | 3.40 | 0.0007 |
| **cigday_l1** | 0.02869 | 0.00406 | 7.07 | <.0001 |
| **drinksday_l1_1** | 0.23370 | 0.18007 | 1.30 | 0.1944 |
| **drinksday_l1_2** | 0.07825 | 0.17148 | 0.46 | 0.6482 |
| **drinksday_l1_3** | 0.01212 | 0.17286 | 0.07 | 0.9441 |
| **bmi_l1** | 0.80464 | 0.01001 | 80.38 | <.0001 |
| **dm_l1** | -0.82520 | 0.22265 | -3.71 | 0.0002 |
| **tsdm_l1_inter** | 0.16204 | 0.12626 | 1.28 | 0.1994 |
| **sbp_l1** | -0.00192 | 0.00168 | -1.14 | 0.2549 |
| **ldlf_l1** | -0.00193 | 0.00089884 | -2.15 | 0.0317 |
| **bpmed_l1** | 0.10287 | 0.07493 | 1.37 | 0.1698 |
| **cigday** | -0.04009 | 0.00452 | -8.87 | <.0001 |
| **drinksday_1** | -0.24644 | 0.18157 | -1.36 | 0.1747 |
| **drinksday_2** | -0.14544 | 0.17411 | -0.84 | 0.4036 |
| **drinksday_3** | -0.13145 | 0.17670 | -0.74 | 0.4570 |

1. Logistic model to estimate the probability of developing diabetes among those without diabetes

| **Parameter** | **Estimate** | **Standard Error** | **Wald Chi-Square** | **Pr > ChiSq** |
| --- | --- | --- | --- | --- |
| **Intercept** | -13.9690 | 2.7357 | 26.0740 | <.0001 |
| **sex** | -0.4258 | 0.1741 | 5.9825 | 0.0144 |
| **age_bl** | 0.1511 | 0.0975 | 2.4028 | 0.1211 |
| **ageage_bl** | -0.00126 | 0.000927 | 1.8414 | 0.1748 |
| **edu1** | 0.5354 | 0.3770 | 2.0171 | 0.1555 |
| **edu2** | 0.2370 | 0.2837 | 0.6983 | 0.4034 |
| **edu3** | -0.0162 | 0.3301 | 0.0024 | 0.9609 |
| **mastat1** | -0.4181 | 0.5125 | 0.6656 | 0.4146 |
| **mastat2** | 0.1740 | 0.2453 | 0.5031 | 0.4781 |
| **eversmok** | 0.1559 | 0.1902 | 0.6718 | 0.4124 |
| **cigday_prebl1** | -1.3088 | 1.0371 | 1.5926 | 0.2070 |
| **cigday_prebl2** | -0.2060 | 0.4029 | 0.2614 | 0.6092 |
| **cigday_prebl3** | 0.4772 | 0.2533 | 3.5496 | 0.0596 |
| **drinksday_prebl1** | 0.6670 | 0.5388 | 1.5326 | 0.2157 |
| **drinksday_prebl2** | 0.6836 | 0.5103 | 1.7946 | 0.1804 |
| **drinksday_prebl3** | 0.6520 | 0.5070 | 1.6537 | 0.1985 |
| **bmi_prebl** | -0.0512 | 0.0354 | 2.0973 | 0.1476 |
| **dm_prebl** | 0 | . | . | . |
| **sbp_prebl** | -0.00680 | 0.00688 | 0.9783 | 0.3226 |
| **ldlf_prebl** | 0.00828 | 0.00289 | 8.2411 | 0.0041 |
| **bpmed_prebl** | 0.0251 | 0.2438 | 0.0106 | 0.9181 |
| **antichol_prebl** | 0.4512 | 0.6609 | 0.4661 | 0.4948 |
| **exam_1** | 0 | . | . | . |
| **exam_2** | 0.2493 | 0.2160 | 1.3324 | 0.2484 |
| **exam_3** | 0.3904 | 0.2018 | 3.7432 | 0.0530 |
| **cigday_l1** | -0.00445 | 0.0143 | 0.0964 | 0.7562 |
| **drinksday_l1_1** | -0.3543 | 0.6256 | 0.3208 | 0.5712 |
| **drinksday_l1_2** | -0.0925 | 0.5933 | 0.0243 | 0.8760 |
| **drinksday_l1_3** | -0.1651 | 0.6036 | 0.0748 | 0.7845 |
| **bmi_l1** | 0.0819 | 0.0417 | 3.8497 | 0.0498 |
| **sbp_l1** | 0.0146 | 0.00541 | 7.3218 | 0.0068 |
| **ldlf_l1** | -0.00472 | 0.00320 | 2.1767 | 0.1401 |
| **bpmed_l1** | 0.5216 | 0.2249 | 5.3816 | 0.0203 |
| **cigday** | 0.00993 | 0.0156 | 0.4069 | 0.5236 |
| **drinksday_1** | 0.0826 | 0.6063 | 0.0185 | 0.8917 |
| **drinksday_2** | -0.3503 | 0.5820 | 0.3621 | 0.5473 |
| **drinksday_3** | -0.2705 | 0.5961 | 0.2059 | 0.6500 |
| **bmi** | 0.0998 | 0.0361 | 7.6407 | 0.0057 |

1. Linear model to estimate systolic blood pressure

| **Variable** | **Parameter Estimate** | **Standard Error** | **t Value** | **Pr > \|t\|** |
| --- | --- | --- | --- | --- |
| **Intercept** | 8.07892 | 4.30910 | 1.87 | 0.0609 |
| **sex** | -0.39782 | 0.33342 | -1.19 | 0.2329 |
| **age_bl** | 0.58536 | 0.15322 | 3.82 | 0.0001 |
| **ageage_bl** | -0.00377 | 0.00151 | -2.49 | 0.0127 |
| **edu1** | 0.37591 | 0.75175 | 0.50 | 0.6171 |
| **edu2** | 0.43636 | 0.47164 | 0.93 | 0.3549 |
| **edu3** | -0.30979 | 0.53003 | -0.58 | 0.5589 |
| **mastat1** | 0.31321 | 0.71457 | 0.44 | 0.6612 |
| **mastat2** | 0.42421 | 0.46260 | 0.92 | 0.3592 |
| **eversmok** | -0.19632 | 0.34692 | -0.57 | 0.5715 |
| **cigday_prebl1** | -0.24390 | 1.05671 | -0.23 | 0.8175 |
| **cigday_prebl2** | 0.82656 | 0.78017 | 1.06 | 0.2894 |
| **cigday_prebl3** | -0.55702 | 0.54653 | -1.02 | 0.3081 |
| **drinksday_prebl1** | 1.02487 | 0.97718 | 1.05 | 0.2943 |
| **drinksday_prebl2** | 0.32782 | 0.91655 | 0.36 | 0.7206 |
| **drinksday_prebl3** | -0.20568 | 0.92759 | -0.22 | 0.8245 |
| **bmi_prebl** | -0.30204 | 0.07890 | -3.83 | 0.0001 |
| **dm_prebl** | 2.87391 | 1.32285 | 2.17 | 0.0298 |
| **sbp_prebl** | 0.27595 | 0.01403 | 19.67 | <.0001 |
| **ldlf_prebl** | -0.00837 | 0.00606 | -1.38 | 0.1673 |
| **bpmed_prebl** | 1.96328 | 0.57021 | 3.44 | 0.0006 |
| **antichol_prebl** | -2.93990 | 1.96299 | -1.50 | 0.1343 |
| **exam_1** | 0 | . | . | . |
| **exam_2** | -0.57833 | 0.38960 | -1.48 | 0.1377 |
| **exam_3** | 1.59144 | 0.38430 | 4.14 | <.0001 |
| **cigday_l1** | -0.04017 | 0.02819 | -1.43 | 0.1542 |
| **drinksday_l1_1** | 2.59874 | 1.24720 | 2.08 | 0.0372 |
| **drinksday_l1_2** | 1.73717 | 1.18751 | 1.46 | 0.1435 |
| **drinksday_l1_3** | 1.82696 | 1.19704 | 1.53 | 0.1270 |
| **bmi_l1** | -0.73227 | 0.09536 | -7.68 | <.0001 |
| **dm** | 2.28574 | 1.30102 | 1.76 | 0.0790 |
| **tsdm_inter** | -0.83872 | 0.60821 | -1.38 | 0.1679 |
| **sbp_l1** | 0.48809 | 0.01166 | 41.86 | <.0001 |
| **ldlf_l1** | 0.01934 | 0.00623 | 3.11 | 0.0019 |
| **bpmed_l1** | -1.44197 | 0.51882 | -2.78 | 0.0055 |
| **cigday** | 0.01830 | 0.03147 | 0.58 | 0.5609 |
| **drinksday_1** | -4.44605 | 1.25727 | -3.54 | 0.0004 |
| **drinksday_2** | -3.35272 | 1.20535 | -2.78 | 0.0054 |
| **drinksday_3** | -2.77392 | 1.22354 | -2.27 | 0.0234 |
| **bmi** | 1.13122 | 0.08143 | 13.89 | <.0001 |

1. Linear model to estimate LDL-cholesterol

| **Variable** | **Parameter Estimate** | **Standard Error** | **t Value** | **Pr > \|t\|** |
| --- | --- | --- | --- | --- |
| **Intercept** | 19.73502 | 7.71183 | 2.56 | 0.0105 |
| **sex** | 0.61410 | 0.59663 | 1.03 | 0.3034 |
| **age_bl** | 0.46096 | 0.27443 | 1.68 | 0.0931 |
| **ageage_bl** | -0.00609 | 0.00271 | -2.25 | 0.0246 |
| **edu1** | -1.98756 | 1.34507 | -1.48 | 0.1395 |
| **edu2** | -0.14252 | 0.84392 | -0.17 | 0.8659 |
| **edu3** | 0.96955 | 0.94836 | 1.02 | 0.3067 |
| **mastat1** | 0.64047 | 1.27855 | 0.50 | 0.6164 |
| **mastat2** | -0.03951 | 0.82775 | -0.05 | 0.9619 |
| **eversmok** | 0.51399 | 0.62073 | 0.83 | 0.4077 |
| **cigday_prebl1** | -0.77271 | 1.89071 | -0.41 | 0.6828 |
| **cigday_prebl2** | -3.26269 | 1.39601 | -2.34 | 0.0195 |
| **cigday_prebl3** | -0.21378 | 0.97794 | -0.22 | 0.8270 |
| **drinksday_prebl1** | -1.16357 | 1.74853 | -0.67 | 0.5058 |
| **drinksday_prebl2** | -0.75408 | 1.63994 | -0.46 | 0.6457 |
| **drinksday_prebl3** | -1.11876 | 1.65968 | -0.67 | 0.5003 |
| **bmi_prebl** | -0.43565 | 0.14131 | -3.08 | 0.0021 |
| **dm_prebl** | 5.55663 | 2.36765 | 2.35 | 0.0190 |
| **sbp_prebl** | -0.08261 | 0.02577 | -3.21 | 0.0014 |
| **ldlf_prebl** | 0.22199 | 0.01085 | 20.47 | <.0001 |
| **bpmed_prebl** | -1.59971 | 1.02107 | -1.57 | 0.1172 |
| **antichol_prebl** | -3.83156 | 3.51279 | -1.09 | 0.2754 |
| **exam_1** | 0 | . | . | . |
| **exam_2** | 2.55224 | 0.69719 | 3.66 | 0.0003 |
| **exam_3** | 5.58085 | 0.68842 | 8.11 | <.0001 |
| **cigday_l1** | -0.09032 | 0.05044 | -1.79 | 0.0734 |
| **drinksday_l1_1** | -0.18558 | 2.23219 | -0.08 | 0.9337 |
| **drinksday_l1_2** | -0.46130 | 2.12504 | -0.22 | 0.8282 |
| **drinksday_l1_3** | -0.20061 | 2.14212 | -0.09 | 0.9254 |
| **bmi_l1** | -0.84348 | 0.17132 | -4.92 | <.0001 |
| **dm** | -6.45123 | 2.32831 | -2.77 | 0.0056 |
| **tsdm_inter** | 0.45364 | 1.08837 | 0.42 | 0.6768 |
| **sbp_l1** | 0.03341 | 0.02326 | 1.44 | 0.1509 |
| **ldlf_l1** | 0.53232 | 0.01115 | 47.74 | <.0001 |
| **bpmed_l1** | -2.86777 | 0.92878 | -3.09 | 0.0020 |
| **cigday** | 0.11872 | 0.05631 | 2.11 | 0.0350 |
| **drinksday_1** | 1.58415 | 2.25148 | 0.70 | 0.4817 |
| **drinksday_2** | 1.30691 | 2.15779 | 0.61 | 0.5448 |
| **drinksday_3** | 0.04459 | 2.18996 | 0.02 | 0.9838 |
| **bmi** | 1.13206 | 0.14763 | 7.67 | <.0001 |
| **sbp** | 0.04964 | 0.02106 | 2.36 | 0.0184 |

1. Logistic model to estimate the probability of taking blood pressure medication

| **Parameter** | **Estimate** | **Standard Error** | **Wald Chi-Square** | **Pr > ChiSq** |
| --- | --- | --- | --- | --- |
| **Intercept** | -14.9398 | 1.5504 | 92.8604 | <.0001 |
| **sex** | -0.0773 | 0.0997 | 0.6015 | 0.4380 |
| **age_bl** | 0.1669 | 0.0553 | 9.1108 | 0.0025 |
| **ageage_bl** | -0.00160 | 0.000528 | 9.2307 | 0.0024 |
| **edu1** | 0.1209 | 0.2219 | 0.2967 | 0.5859 |
| **edu2** | 0.1950 | 0.1486 | 1.7216 | 0.1895 |
| **edu3** | -0.0540 | 0.1701 | 0.1009 | 0.7507 |
| **mastat1** | -0.3468 | 0.2322 | 2.2317 | 0.1352 |
| **mastat2** | -0.2711 | 0.1359 | 3.9778 | 0.0461 |
| **eversmok** | 0.1381 | 0.1044 | 1.7486 | 0.1861 |
| **cigday_prebl1** | 0.0389 | 0.3429 | 0.0129 | 0.9097 |
| **cigday_prebl2** | 0.2899 | 0.2224 | 1.6990 | 0.1924 |
| **cigday_prebl3** | 0.1835 | 0.1693 | 1.1754 | 0.2783 |
| **drinksday_prebl1** | 0.6013 | 0.2810 | 4.5797 | 0.0324 |
| **drinksday_prebl2** | 0.3100 | 0.2605 | 1.4152 | 0.2342 |
| **drinksday_prebl3** | 0.2611 | 0.2637 | 0.9799 | 0.3222 |
| **bmi_prebl** | -0.0584 | 0.0222 | 6.8862 | 0.0087 |
| **dm_prebl** | 0.2574 | 0.3469 | 0.5504 | 0.4582 |
| **sbp_prebl** | 0.0319 | 0.00404 | 62.5827 | <.0001 |
| **ldlf_prebl** | 0.00198 | 0.00180 | 1.2086 | 0.2716 |
| **bpmed_prebl** | 0.9604 | 0.1406 | 46.6596 | <.0001 |
| **antichol_prebl** | 0.0902 | 0.5265 | 0.0293 | 0.8640 |
| **exam_1** | 0 | . | . | . |
| **exam_2** | -1.1703 | 0.1272 | 84.5898 | <.0001 |
| **exam_3** | 0.1426 | 0.1084 | 1.7288 | 0.1886 |
| **cigday_l1** | -0.00273 | 0.00864 | 0.0999 | 0.7519 |
| **drinksday_l1_1** | -0.1682 | 0.3689 | 0.2079 | 0.6484 |
| **drinksday_l1_2** | -0.0654 | 0.3489 | 0.0351 | 0.8513 |
| **drinksday_l1_3** | 0.0339 | 0.3489 | 0.0094 | 0.9227 |
| **bmi_l1** | -0.0575 | 0.0271 | 4.4975 | 0.0339 |
| **dm** | -0.5970 | 0.3363 | 3.1506 | 0.0759 |
| **tsdm_inter** | 0.4619 | 0.1565 | 8.7140 | 0.0032 |
| **sbp_l1** | 0.0529 | 0.00360 | 215.8098 | <.0001 |
| **ldlf_l1** | 0.000696 | 0.00211 | 0.1088 | 0.7416 |
| **bpmed_l1** | 3.8073 | 0.1266 | 904.3450 | <.0001 |
| **cigday** | -0.0203 | 0.00999 | 4.1487 | 0.0417 |
| **drinksday_1** | -0.1619 | 0.3694 | 0.1921 | 0.6612 |
| **drinksday_2** | -0.3181 | 0.3519 | 0.8171 | 0.3660 |
| **drinksday_3** | 0.1039 | 0.3550 | 0.0856 | 0.7699 |
| **bmi** | 0.1211 | 0.0236 | 26.2937 | <.0001 |
| **sbp** | -0.0184 | 0.00326 | 31.7920 | <.0001 |
| **ldlf** | -0.00343 | 0.00193 | 3.1461 | 0.0761 |
