# Supplementary material for: Long-term moderately elevated LDL-cholesterol and blood pressure and risk of coronary heart disease
Source: PLoS One. 2018 Jul 30;13(7):e0200017. doi: 10.1371/journal.pone.0200017 (PMC6066205; doi:10.1371/journal.pone.0200017)
Supplement: S3 Table — (DOCX) [file pone.0200017.s003.docx]

**S3 Table. Coefficients of regressions used in the simulations on systolic blood pressure**^a,b,c^

^a^ For the list of code names, see S1 Table. For variables with more than two categories, the highest category is used as the reference.

^b^ The suffixes in the variable names are as follows: the numerical suffixes represent the categories of the values of the covariate as defined in the footnotes of S1 Table; l1 indicates the value lagged for one examination cycle

^c^ *tsdm_l1_inter* is the product of the lagged value of *dm* (diabetes) variable and the cumulative sum of the *dm* variable from the baseline to the preceding examination cycle.

1. Logistic model to estimate the probability of coronary heart disease

| **Parameter** | **Estimate** | **Standard Error** | **Wald Chi-Square** | **Pr > ChiSq** |
| --- | --- | --- | --- | --- |
| **Intercept** | -10.3357 | 2.7181 | 14.4599 | 0.0001 |
| **sex** | -1.0130 | 0.1643 | 38.0028 | <.0001 |
| **age_bl** | 0.1713 | 0.0975 | 3.0891 | 0.0788 |
| **ageage_bl** | -0.00132 | 0.000909 | 2.1129 | 0.1461 |
| **edu1** | 0.3011 | 0.3278 | 0.8437 | 0.3584 |
| **edu2** | 0.1461 | 0.2465 | 0.3514 | 0.5533 |
| **edu3** | 0.3137 | 0.2724 | 1.3266 | 0.2494 |
| **mastat1** | -0.5939 | 0.3972 | 2.2359 | 0.1348 |
| **mastat2** | -0.4561 | 0.2035 | 5.0228 | 0.0250 |
| **eversmok** | 0.0639 | 0.1744 | 0.1344 | 0.7139 |
| **cigday_prebl0** | -0.4822 | 0.2913 | 2.7412 | 0.0978 |
| **cigday_prebl1** | -0.3028 | 0.6426 | 0.2220 | 0.6375 |
| **cigday_prebl2** | -0.6904 | 0.4827 | 2.0457 | 0.1526 |
| **cigday_prebl3** | -0.3985 | 0.3197 | 1.5543 | 0.2125 |
| **drinksday_prebl1** | 0.1081 | 0.3734 | 0.0837 | 0.7723 |
| **drinksday_prebl2** | 0.0508 | 0.3433 | 0.0219 | 0.8824 |
| **drinksday_prebl3** | 0.3120 | 0.3412 | 0.8362 | 0.3605 |
| **bmi_prebl** | 0.1358 | 0.0310 | 19.2343 | <.0001 |
| **dm_prebl** | 0.2573 | 0.3409 | 0.5697 | 0.4504 |
| **sbp_prebl** | -0.00460 | 0.00565 | 0.6628 | 0.4156 |
| **ldlf_prebl** | 0.00565 | 0.00262 | 4.6364 | 0.0313 |
| **antichol_prebl** | 0.8189 | 0.5596 | 2.1420 | 0.1433 |
| **bpmed_prebl** | 0.3523 | 0.1790 | 3.8750 | 0.0490 |
| **exam_1** | -0.5379 | 0.2217 | 5.8891 | 0.0152 |
| **exam_2** | -0.3422 | 0.2095 | 2.6683 | 0.1024 |
| **exam_3** | -0.1655 | 0.1973 | 0.7033 | 0.4017 |
| **cigday** | 0.00917 | 0.00931 | 0.9710 | 0.3244 |
| **drinksday_1** | -0.1510 | 0.4040 | 0.1397 | 0.7085 |
| **drinksday_2** | -0.3328 | 0.3794 | 0.7697 | 0.3803 |
| **drinksday_3** | -0.6566 | 0.4048 | 2.6307 | 0.1048 |
| **bmi** | -0.1067 | 0.0295 | 13.0818 | 0.0003 |
| **dm** | 0.3957 | 0.3907 | 1.0258 | 0.3112 |
| **tsdm_inter** | 0.0809 | 0.1632 | 0.2456 | 0.6202 |
| **sbp** | 0.0171 | 0.00440 | 15.0439 | 0.0001 |
| **ldlf** | 0.00324 | 0.00268 | 1.4666 | 0.2259 |
| **antichol** | 0.2107 | 0.2295 | 0.8429 | 0.3586 |

1. Logistic model to estimate the probability of death from a cause other than coronary heart disease

| **Parameter** | **Estimate** | **Standard Error** | **Wald Chi-Square** | **Pr > ChiSq** |
| --- | --- | --- | --- | --- |
| **Intercept** | -6.1307 | 3.4506 | 3.1567 | 0.0756 |
| **sex** | -0.5931 | 0.1833 | 10.4678 | 0.0012 |
| **age_bl** | 0.1345 | 0.1221 | 1.2130 | 0.2707 |
| **ageage_bl** | -0.00042 | 0.00111 | 0.1432 | 0.7051 |
| **edu1** | 0.5929 | 0.3568 | 2.7613 | 0.0966 |
| **edu2** | 0.1450 | 0.2956 | 0.2407 | 0.6237 |
| **edu3** | -0.2828 | 0.3595 | 0.6188 | 0.4315 |
| **mastat1** | -0.4105 | 0.4681 | 0.7691 | 0.3805 |
| **mastat2** | -0.3327 | 0.2310 | 2.0749 | 0.1497 |
| **eversmok** | 0.2313 | 0.2138 | 1.1694 | 0.2795 |
| **cigday_prebl0** | -0.9614 | 0.3127 | 9.4507 | 0.0021 |
| **cigday_prebl1** | -0.9419 | 0.7660 | 1.5121 | 0.2188 |
| **cigday_prebl2** | -0.7041 | 0.4901 | 2.0638 | 0.1508 |
| **cigday_prebl3** | -0.4133 | 0.3276 | 1.5921 | 0.2070 |
| **drinksday_prebl1** | -0.2761 | 0.4194 | 0.4335 | 0.5103 |
| **drinksday_prebl2** | -0.0241 | 0.3768 | 0.0041 | 0.9490 |
| **drinksday_prebl3** | -0.1135 | 0.3757 | 0.0912 | 0.7626 |
| **bmi_prebl** | 0.1842 | 0.0359 | 26.3310 | <.0001 |
| **dm_prebl** | 0.1068 | 0.4504 | 0.0563 | 0.8125 |
| **sbp_prebl** | -0.00069 | 0.00630 | 0.0119 | 0.9131 |
| **ldlf_prebl** | 0.000416 | 0.00326 | 0.0163 | 0.8983 |
| **antichol_prebl** | -0.3362 | 1.0479 | 0.1029 | 0.7483 |
| **bpmed_prebl** | 0.2430 | 0.2153 | 1.2747 | 0.2589 |
| **exam_1** | -1.1173 | 0.2577 | 18.8016 | <.0001 |
| **exam_2** | -0.6000 | 0.2239 | 7.1833 | 0.0074 |
| **exam_3** | -0.5887 | 0.2268 | 6.7389 | 0.0094 |
| **cigday** | -0.00030 | 0.0106 | 0.0008 | 0.9772 |
| **drinksday_1** | -0.4158 | 0.4553 | 0.8341 | 0.3611 |
| **drinksday_2** | -0.6653 | 0.4288 | 2.4074 | 0.1208 |
| **drinksday_3** | -0.5943 | 0.4403 | 1.8217 | 0.1771 |
| **bmi** | -0.1908 | 0.0349 | 29.8702 | <.0001 |
| **dm** | 0.8107 | 0.4927 | 2.7078 | 0.0999 |
| **tsdm_inter** | -0.2081 | 0.2125 | 0.9595 | 0.3273 |
| **sbp** | -0.00249 | 0.00545 | 0.2081 | 0.6483 |
| **ldlf** | -0.00452 | 0.00333 | 1.8416 | 0.1748 |
| **antichol** | -0.2321 | 0.3082 | 0.5671 | 0.4514 |

1. Logistic model to estimate the probability of smoking

| **Parameter** | **Estimate** | **Standard Error** | **Wald Chi-Square** | **Pr > ChiSq** |
| --- | --- | --- | --- | --- |
| **Intercept** | 0.5672 | 1.5644 | 0.1314 | 0.7169 |
| **sex** | 0.3526 | 0.1240 | 8.0806 | 0.0045 |
| **age_bl** | -0.1270 | 0.0547 | 5.3879 | 0.0203 |
| **ageage_bl** | 0.000922 | 0.000557 | 2.7406 | 0.0978 |
| **edu1** | 0.4709 | 0.2764 | 2.9022 | 0.0885 |
| **edu2** | 0.0754 | 0.1993 | 0.1431 | 0.7053 |
| **edu3** | -0.0792 | 0.2261 | 0.1227 | 0.7261 |
| **mastat1** | 0.2150 | 0.2336 | 0.8476 | 0.3572 |
| **mastat2** | -0.1834 | 0.1539 | 1.4217 | 0.2331 |
| **eversmok** | 2.4347 | 0.4285 | 32.2808 | <.0001 |
| **cigday_prebl0** | -1.9269 | 0.1936 | 99.0666 | <.0001 |
| **cigday_prebl1** | -0.1177 | 0.2682 | 0.1925 | 0.6608 |
| **cigday_prebl2** | 0.6589 | 0.1929 | 11.6646 | 0.0006 |
| **cigday_prebl3** | 0.6011 | 0.1533 | 15.3817 | <.0001 |
| **drinksday_prebl1** | -0.0237 | 0.3315 | 0.0051 | 0.9430 |
| **drinksday_prebl2** | -0.0591 | 0.3076 | 0.0369 | 0.8478 |
| **drinksday_prebl3** | 0.0547 | 0.3089 | 0.0314 | 0.8594 |
| **bmi_prebl** | 0.1144 | 0.0276 | 17.1748 | <.0001 |
| **dm_prebl** | -0.0218 | 0.7992 | 0.0007 | 0.9783 |
| **sbp_prebl** | -0.00010 | 0.00513 | 0.0004 | 0.9837 |
| **ldlf_prebl** | -0.00226 | 0.00237 | 0.9076 | 0.3408 |
| **antichol_prebl** | 0.5326 | 0.7166 | 0.5525 | 0.4573 |
| **bpmed_prebl** | -0.0959 | 0.2056 | 0.2177 | 0.6408 |
| **exam_1** | 0 | . | . | . |
| **exam_2** | 0.1852 | 0.1420 | 1.7000 | 0.1923 |
| **exam_3** | -0.0607 | 0.1406 | 0.1865 | 0.6659 |
| **cigday_l1** | 0.1315 | 0.00622 | 447.3040 | <.0001 |
| **drinksday_l1_1** | -0.6904 | 0.3726 | 3.4336 | 0.0639 |
| **drinksday_l1_2** | -0.7234 | 0.3549 | 4.1540 | 0.0415 |
| **drinksday_l1_3** | -0.4705 | 0.3639 | 1.6716 | 0.1960 |
| **bmi_l1** | -0.1188 | 0.0248 | 22.9737 | <.0001 |
| **dm_l1** | -0.0470 | 0.6317 | 0.0055 | 0.9407 |
| **tsdm_l1_inter** | 0.00899 | 0.3829 | 0.0006 | 0.9813 |
| **sbp_l1** | -0.00310 | 0.00443 | 0.4899 | 0.4840 |
| **ldlf_l1** | 0.00189 | 0.00228 | 0.6818 | 0.4090 |
| **antichol_l1** | -0.0561 | 0.2870 | 0.0382 | 0.8450 |

1. Log-linear model to estimate the number of cigarettes smoked per day among smokers

| **Variable** | **Parameter Estimate** | **Standard Error** | **t Value** | **Pr > \|t\|** |
| --- | --- | --- | --- | --- |
| **Intercept** | 2.05684 | 0.54003 | 3.81 | 0.0001 |
| **sex** | -0.03723 | 0.03900 | -0.95 | 0.3400 |
| **age_bl** | 0.01011 | 0.01740 | 0.58 | 0.5615 |
| **ageage_bl** | -0.00011706 | 0.00017942 | -0.65 | 0.5142 |
| **edu1** | 0.31875 | 0.09518 | 3.35 | 0.0008 |
| **edu2** | 0.27209 | 0.07241 | 3.76 | 0.0002 |
| **edu3** | 0.20279 | 0.08243 | 2.46 | 0.0140 |
| **mastat1** | -0.04683 | 0.06900 | -0.68 | 0.4975 |
| **mastat2** | -0.02520 | 0.04718 | -0.53 | 0.5934 |
| **eversmok** | -0.42931 | 0.24751 | -1.73 | 0.0831 |
| **cigday_prebl0** | -0.43645 | 0.08009 | -5.45 | <.0001 |
| **cigday_prebl1** | -1.09931 | 0.11107 | -9.90 | <.0001 |
| **cigday_prebl2** | -0.42794 | 0.07035 | -6.08 | <.0001 |
| **cigday_prebl3** | -0.07140 | 0.04494 | -1.59 | 0.1124 |
| **drinksday_prebl1** | 0.04394 | 0.09114 | 0.48 | 0.6298 |
| **drinksday_prebl2** | -0.00788 | 0.08213 | -0.10 | 0.9236 |
| **drinksday_prebl3** | 0.02772 | 0.08217 | 0.34 | 0.7359 |
| **bmi_prebl** | 0.01917 | 0.00884 | 2.17 | 0.0303 |
| **dm_prebl** | 0.18088 | 0.20997 | 0.86 | 0.3892 |
| **sbp_prebl** | -0.00037770 | 0.00161 | -0.24 | 0.8140 |
| **ldlf_prebl** | 0.00009946 | 0.00077738 | 0.13 | 0.8982 |
| **antichol_prebl** | 0.03049 | 0.27610 | 0.11 | 0.9121 |
| **bpmed_prebl** | 0.02951 | 0.07029 | 0.42 | 0.6746 |
| **exam_1** | 0 | . | . | . |
| **exam_2** | 0.07417 | 0.04585 | 1.62 | 0.1060 |
| **exam_3** | 0.00243 | 0.04731 | 0.05 | 0.9591 |
| **cigday_l1** | 0.03428 | 0.00182 | 18.79 | <.0001 |
| **drinksday_l1_1** | -0.05107 | 0.09656 | -0.53 | 0.5970 |
| **drinksday_l1_2** | -0.13405 | 0.08918 | -1.50 | 0.1331 |
| **drinksday_l1_3** | -0.03142 | 0.09260 | -0.34 | 0.7344 |
| **bmi_l1** | -0.01133 | 0.00804 | -1.41 | 0.1592 |
| **dm_l1** | 0.03775 | 0.20474 | 0.18 | 0.8538 |
| **tsdm_l1_inter** | -0.06095 | 0.11320 | -0.54 | 0.5904 |
| **sbp_l1** | 0.00123 | 0.00144 | 0.85 | 0.3946 |
| **ldlf_l1** | -0.00066029 | 0.00070849 | -0.93 | 0.3516 |
| **antichol_l1** | -0.09390 | 0.10178 | -0.92 | 0.3565 |

1. Logistic model to estimate the probability of starting to drink alcohol among those who do not drink

| **Parameter** | **Estimate** | **Standard Error** | **Wald Chi-Square** | **Pr > ChiSq** |
| --- | --- | --- | --- | --- |
| **Intercept** | -0.4781 | 1.4744 | 0.1051 | 0.7458 |
| **sex** | 0.1498 | 0.1196 | 1.5680 | 0.2105 |
| **age_bl** | -0.0327 | 0.0521 | 0.3939 | 0.5302 |
| **ageage_bl** | 0.000106 | 0.000518 | 0.0421 | 0.8374 |
| **edu1** | -0.6114 | 0.2801 | 4.7647 | 0.0290 |
| **edu2** | -0.5010 | 0.1791 | 7.8284 | 0.0051 |
| **edu3** | -0.0684 | 0.2004 | 0.1166 | 0.7327 |
| **mastat1** | 0.3788 | 0.2375 | 2.5425 | 0.1108 |
| **mastat2** | 0.3735 | 0.1693 | 4.8644 | 0.0274 |
| **eversmok** | 0.2981 | 0.1252 | 5.6725 | 0.0172 |
| **cigday_prebl0** | 0.7045 | 0.2692 | 6.8489 | 0.0089 |
| **cigday_prebl1** | 1.1925 | 0.5017 | 5.6495 | 0.0175 |
| **cigday_prebl2** | 0.7019 | 0.3332 | 4.4382 | 0.0351 |
| **cigday_prebl3** | 0.6885 | 0.2544 | 7.3249 | 0.0068 |
| **drinksday_prebl1** | -1.0304 | 0.4848 | 4.5173 | 0.0336 |
| **drinksday_prebl2** | 0.1972 | 0.4833 | 0.1665 | 0.6832 |
| **drinksday_prebl3** | -0.2062 | 0.5556 | 0.1378 | 0.7105 |
| **bmi_prebl** | -0.0341 | 0.0253 | 1.8139 | 0.1780 |
| **dm_prebl** | -0.00215 | 0.6681 | 0.0000 | 0.9974 |
| **sbp_prebl** | -0.00655 | 0.00484 | 1.8335 | 0.1757 |
| **ldlf_prebl** | 0.00656 | 0.00220 | 8.8825 | 0.0029 |
| **antichol_prebl** | 0.7506 | 0.6124 | 1.5022 | 0.2203 |
| **bpmed_prebl** | -0.0468 | 0.1728 | 0.0733 | 0.7866 |
| **exam_1** | 0 | . | . | . |
| **exam_2** | 0.1549 | 0.1299 | 1.4212 | 0.2332 |
| **exam_3** | -0.5201 | 0.1371 | 14.3988 | 0.0001 |
| **cigday_l1** | 0.00672 | 0.0109 | 0.3774 | 0.5390 |
| **drinksday_l1_1** | 0 | . | . | . |
| **drinksday_l1_2** | 0 | . | . | . |
| **drinksday_l1_3** | 0 | . | . | . |
| **bmi_l1** | 0.0148 | 0.0228 | 0.4202 | 0.5169 |
| **dm_l1** | 0.0733 | 0.5640 | 0.0169 | 0.8966 |
| **tsdm_l1_inter** | -0.3463 | 0.3384 | 1.0474 | 0.3061 |
| **sbp_l1** | 0.00659 | 0.00416 | 2.5188 | 0.1125 |
| **ldlf_l1** | -0.00167 | 0.00225 | 0.5493 | 0.4586 |
| **antichol_l1** | 0.1654 | 0.2364 | 0.4897 | 0.4840 |
| **cigday** | 0.00301 | 0.0116 | 0.0679 | 0.7944 |

1. Logistic model to estimate the probability of continuing to drink alcohol among those who drink

| **Parameter** | **Estimate** | **Standard Error** | **Wald Chi-Square** | **Pr > ChiSq** |
| --- | --- | --- | --- | --- |
| **Intercept** | 5.0155 | 1.3085 | 14.6924 | 0.0001 |
| **sex** | -0.3874 | 0.1021 | 14.3828 | 0.0001 |
| **age_bl** | 0.0173 | 0.0463 | 0.1394 | 0.7088 |
| **ageage_bl** | -0.00037 | 0.000454 | 0.6496 | 0.4203 |
| **edu1** | -0.3129 | 0.2339 | 1.7906 | 0.1809 |
| **edu2** | -0.3797 | 0.1515 | 6.2802 | 0.0122 |
| **edu3** | -0.1717 | 0.1717 | 1.0003 | 0.3172 |
| **mastat1** | -0.5897 | 0.2057 | 8.2223 | 0.0041 |
| **mastat2** | 0.00256 | 0.1406 | 0.0003 | 0.9855 |
| **eversmok** | 0.2735 | 0.1078 | 6.4416 | 0.0111 |
| **cigday_prebl0** | 0.0770 | 0.2274 | 0.1146 | 0.7349 |
| **cigday_prebl1** | 0.0719 | 0.3776 | 0.0363 | 0.8490 |
| **cigday_prebl2** | 0.0602 | 0.3016 | 0.0398 | 0.8418 |
| **cigday_prebl3** | -0.3523 | 0.2095 | 2.8290 | 0.0926 |
| **drinksday_prebl1** | -1.6969 | 0.3025 | 31.4702 | <.0001 |
| **drinksday_prebl2** | -0.2636 | 0.2916 | 0.8169 | 0.3661 |
| **drinksday_prebl3** | 0.5169 | 0.3123 | 2.7383 | 0.0980 |
| **bmi_prebl** | -0.0144 | 0.0230 | 0.3894 | 0.5326 |
| **dm_prebl** | 0.6491 | 0.5054 | 1.6498 | 0.1990 |
| **sbp_prebl** | -0.00151 | 0.00407 | 0.1376 | 0.7107 |
| **ldlf_prebl** | 0.00137 | 0.00191 | 0.5126 | 0.4740 |
| **antichol_prebl** | 0.5021 | 0.6083 | 0.6812 | 0.4092 |
| **bpmed_prebl** | 0.0408 | 0.1458 | 0.0782 | 0.7798 |
| **exam_1** | 0 | . | . | . |
| **exam_2** | -0.2040 | 0.1292 | 2.4940 | 0.1143 |
| **exam_3** | -0.6937 | 0.1193 | 33.8240 | <.0001 |
| **cigday_l1** | -0.00541 | 0.00922 | 0.3450 | 0.5569 |
| **drinksday_l1_1** | 0 | . | . | . |
| **drinksday_l1_2** | 0.0640 | 0.3179 | 0.0405 | 0.8405 |
| **drinksday_l1_3** | 1.0332 | 0.3527 | 8.5818 | 0.0034 |
| **bmi_l1** | -0.0202 | 0.0205 | 0.9682 | 0.3251 |
| **dm_l1** | -0.00092 | 0.4431 | 0.0000 | 0.9984 |
| **tsdm_l1_inter** | -0.3380 | 0.2464 | 1.8814 | 0.1702 |
| **sbp_l1** | -0.00264 | 0.00340 | 0.6012 | 0.4381 |
| **ldlf_l1** | -0.00287 | 0.00191 | 2.2472 | 0.1339 |
| **antichol_l1** | -0.2014 | 0.2218 | 0.8246 | 0.3638 |
| **cigday** | 0.000913 | 0.00951 | 0.0092 | 0.9236 |

1. Log-linear model to estimate the number of drinks per day among those drinking alcohol

| **Variable** | **Parameter Estimate** | **Standard Error** | **t Value** | **Pr > \|t\|** |
| --- | --- | --- | --- | --- |
| **Intercept** | 1.78347 | 0.30960 | 5.76 | <.0001 |
| **sex** | -0.21646 | 0.02426 | -8.92 | <.0001 |
| **age_bl** | -0.00331 | 0.01101 | -0.30 | 0.7635 |
| **ageage_bl** | 0.00000704 | 0.00010927 | 0.06 | 0.9486 |
| **edu1** | -0.04319 | 0.05568 | -0.78 | 0.4379 |
| **edu2** | -0.13351 | 0.03214 | -4.15 | <.0001 |
| **edu3** | -0.04636 | 0.03596 | -1.29 | 0.1974 |
| **mastat1** | -0.01023 | 0.05313 | -0.19 | 0.8474 |
| **mastat2** | 0.01930 | 0.03387 | 0.57 | 0.5689 |
| **eversmok** | 0.14804 | 0.02578 | 5.74 | <.0001 |
| **cigday_prebl0** | 0.04727 | 0.05298 | 0.89 | 0.3723 |
| **cigday_prebl1** | 0.09552 | 0.08270 | 1.16 | 0.2481 |
| **cigday_prebl2** | 0.10501 | 0.06912 | 1.52 | 0.1288 |
| **cigday_prebl3** | 0.05967 | 0.05134 | 1.16 | 0.2452 |
| **drinksday_prebl1** | -1.12976 | 0.06655 | -16.98 | <.0001 |
| **drinksday_prebl2** | -0.72644 | 0.05817 | -12.49 | <.0001 |
| **drinksday_prebl3** | -0.20261 | 0.05866 | -3.45 | 0.0006 |
| **bmi_prebl** | -0.01461 | 0.00604 | -2.42 | 0.0156 |
| **dm_prebl** | 0.12251 | 0.15326 | 0.80 | 0.4241 |
| **sbp_prebl** | 0.00077354 | 0.00100 | 0.77 | 0.4398 |
| **ldlf_prebl** | 0.00048745 | 0.00046578 | 1.05 | 0.2954 |
| **antichol_prebl** | -0.08391 | 0.14283 | -0.59 | 0.5569 |
| **bpmed_prebl** | 0.02022 | 0.03686 | 0.55 | 0.5833 |
| **exam_1** | 0 | . | . | . |
| **exam_2** | -0.17959 | 0.02781 | -6.46 | <.0001 |
| **exam_3** | -0.24579 | 0.02786 | -8.82 | <.0001 |
| **cigday_l1** | -0.00354 | 0.00227 | -1.56 | 0.1189 |
| **drinksday_l1_1** | -1.57039 | 0.07870 | -19.95 | <.0001 |
| **drinksday_l1_2** | -0.94891 | 0.07093 | -13.38 | <.0001 |
| **drinksday_l1_3** | -0.21211 | 0.07213 | -2.94 | 0.0033 |
| **bmi_l1** | -0.00211 | 0.00534 | -0.40 | 0.6924 |
| **dm_l1** | -0.34166 | 0.12950 | -2.64 | 0.0084 |
| **tsdm_l1_inter** | 0.09600 | 0.07695 | 1.25 | 0.2122 |
| **sbp_l1** | 0.00137 | 0.00086280 | 1.58 | 0.1137 |
| **ldlf_l1** | -0.00000851 | 0.00046264 | -0.02 | 0.9853 |
| **antichol_l1** | -0.06397 | 0.05562 | -1.15 | 0.2502 |
| **cigday** | 0.00490 | 0.00232 | 2.11 | 0.0348 |

1. Linear model to estimate body mass index

| **Variable** | **Parameter Estimate** | **Standard Error** | **t Value** | **Pr > \|t\|** |
| --- | --- | --- | --- | --- |
| **Intercept** | 2.30666 | 0.62911 | 3.67 | 0.0002 |
| **sex** | 0.23478 | 0.04801 | 4.89 | <.0001 |
| **age_bl** | -0.01990 | 0.02210 | -0.90 | 0.3678 |
| **ageage_bl** | -0.00003057 | 0.00021826 | -0.14 | 0.8886 |
| **edu1** | -0.00990 | 0.10853 | -0.09 | 0.9273 |
| **edu2** | 0.00881 | 0.06807 | 0.13 | 0.8970 |
| **edu3** | 0.02635 | 0.07642 | 0.34 | 0.7303 |
| **mastat1** | 0.02745 | 0.10300 | 0.27 | 0.7898 |
| **mastat2** | -0.21156 | 0.06661 | -3.18 | 0.0015 |
| **eversmok** | 0.05254 | 0.05109 | 1.03 | 0.3038 |
| **cigday_prebl0** | -0.50434 | 0.10433 | -4.83 | <.0001 |
| **cigday_prebl1** | -0.46401 | 0.17593 | -2.64 | 0.0084 |
| **cigday_prebl2** | -0.30436 | 0.13832 | -2.20 | 0.0278 |
| **cigday_prebl3** | -0.09984 | 0.10118 | -0.99 | 0.3238 |
| **drinksday_prebl1** | -0.06121 | 0.14096 | -0.43 | 0.6641 |
| **drinksday_prebl2** | -0.06047 | 0.13223 | -0.46 | 0.6475 |
| **drinksday_prebl3** | -0.18460 | 0.13378 | -1.38 | 0.1677 |
| **bmi_prebl** | 0.21895 | 0.01123 | 19.49 | <.0001 |
| **dm_prebl** | -0.30566 | 0.25371 | -1.20 | 0.2283 |
| **sbp_prebl** | -0.00260 | 0.00195 | -1.33 | 0.1831 |
| **ldlf_prebl** | 0.00079214 | 0.00091887 | 0.86 | 0.3887 |
| **antichol_prebl** | -0.01697 | 0.29292 | -0.06 | 0.9538 |
| **bpmed_prebl** | 0.10907 | 0.07103 | 1.54 | 0.1247 |
| **exam_1** | 0 | . | . | . |
| **exam_2** | 0.18735 | 0.05614 | 3.34 | 0.0009 |
| **exam_3** | 0.19351 | 0.05514 | 3.51 | 0.0005 |
| **cigday_l1** | 0.01998 | 0.00442 | 4.52 | <.0001 |
| **drinksday_l1_1** | 0.21467 | 0.17980 | 1.19 | 0.2325 |
| **drinksday_l1_2** | 0.06278 | 0.17121 | 0.37 | 0.7139 |
| **drinksday_l1_3** | 0.00140 | 0.17257 | 0.01 | 0.9935 |
| **bmi_l1** | 0.79826 | 0.01007 | 79.28 | <.0001 |
| **dm_l1** | -0.83918 | 0.22219 | -3.78 | 0.0002 |
| **tsdm_l1_inter** | 0.16063 | 0.12598 | 1.28 | 0.2023 |
| **sbp_l1** | -0.00216 | 0.00168 | -1.28 | 0.1989 |
| **ldlf_l1** | -0.00149 | 0.00091486 | -1.63 | 0.1027 |
| **antichol_l1** | 0.23950 | 0.10637 | 2.25 | 0.0244 |
| **cigday** | -0.04300 | 0.00455 | -9.44 | <.0001 |
| **drinksday_1** | -0.26910 | 0.18131 | -1.48 | 0.1378 |
| **drinksday_2** | -0.16189 | 0.17384 | -0.93 | 0.3518 |
| **drinksday_3** | -0.15526 | 0.17646 | -0.88 | 0.3790 |

1. Logistic model to estimate the probability of developing diabetes among those without diabetes

| **Parameter** | **Estimate** | **Standard Error** | **Wald Chi-Square** | **Pr > ChiSq** |
| --- | --- | --- | --- | --- |
| **Intercept** | -14.3352 | 2.7713 | 26.7571 | <.0001 |
| **sex** | -0.4199 | 0.1739 | 5.8322 | 0.0157 |
| **age_bl** | 0.1523 | 0.0979 | 2.4193 | 0.1199 |
| **ageage_bl** | -0.00126 | 0.000931 | 1.8444 | 0.1744 |
| **edu1** | 0.5369 | 0.3780 | 2.0173 | 0.1555 |
| **edu2** | 0.2363 | 0.2839 | 0.6926 | 0.4053 |
| **edu3** | -0.0307 | 0.3299 | 0.0086 | 0.9259 |
| **mastat1** | -0.4251 | 0.5125 | 0.6881 | 0.4068 |
| **mastat2** | 0.1314 | 0.2449 | 0.2879 | 0.5915 |
| **eversmok** | 0.1768 | 0.1932 | 0.8376 | 0.3601 |
| **cigday_prebl0** | 0.0883 | 0.3630 | 0.0592 | 0.8077 |
| **cigday_prebl1** | -1.2064 | 1.0746 | 1.2602 | 0.2616 |
| **cigday_prebl2** | -0.1646 | 0.4906 | 0.1125 | 0.7373 |
| **cigday_prebl3** | 0.5662 | 0.3455 | 2.6856 | 0.1013 |
| **drinksday_prebl1** | 0.6804 | 0.5423 | 1.5739 | 0.2096 |
| **drinksday_prebl2** | 0.6844 | 0.5133 | 1.7782 | 0.1824 |
| **drinksday_prebl3** | 0.6765 | 0.5106 | 1.7552 | 0.1852 |
| **bmi_prebl** | -0.0532 | 0.0358 | 2.2127 | 0.1369 |
| **dm_prebl** | 0 | . | . | . |
| **sbp_prebl** | -0.00377 | 0.00669 | 0.3173 | 0.5733 |
| **ldlf_prebl** | 0.00656 | 0.00309 | 4.5148 | 0.0336 |
| **antichol_prebl** | 0.1213 | 0.6836 | 0.0315 | 0.8591 |
| **bpmed_prebl** | 0.3062 | 0.2067 | 2.1933 | 0.1386 |
| **exam_1** | 0 | . | . | . |
| **exam_2** | 0.2373 | 0.2167 | 1.1993 | 0.2735 |
| **exam_3** | 0.3655 | 0.2013 | 3.2945 | 0.0695 |
| **cigday_l1** | -0.00394 | 0.0155 | 0.0649 | 0.7989 |
| **drinksday_l1_1** | -0.3712 | 0.6298 | 0.3473 | 0.5556 |
| **drinksday_l1_2** | -0.0958 | 0.5977 | 0.0257 | 0.8727 |
| **drinksday_l1_3** | -0.1525 | 0.6085 | 0.0628 | 0.8021 |
| **bmi_l1** | 0.0853 | 0.0422 | 4.0960 | 0.0430 |
| **sbp_l1** | 0.0146 | 0.00542 | 7.2527 | 0.0071 |
| **ldlf_l1** | -0.00350 | 0.00328 | 1.1363 | 0.2864 |
| **antichol_l1** | 0.5293 | 0.2856 | 3.4333 | 0.0639 |
| **cigday** | 0.00945 | 0.0158 | 0.3570 | 0.5502 |
| **drinksday_1** | 0.0765 | 0.6092 | 0.0158 | 0.9001 |
| **drinksday_2** | -0.3659 | 0.5847 | 0.3915 | 0.5315 |
| **drinksday_3** | -0.3039 | 0.5995 | 0.2569 | 0.6122 |
| **bmi** | 0.0996 | 0.0366 | 7.4180 | 0.0065 |

1. Linear model to estimate systolic blood pressure

| **Variable** | **Parameter Estimate** | **Standard Error** | **t Value** | **Pr > \|t\|** |
| --- | --- | --- | --- | --- |
| **Intercept** | 10.75587 | 4.37041 | 2.46 | 0.0139 |
| **sex** | -0.37363 | 0.33349 | -1.12 | 0.2626 |
| **age_bl** | 0.58590 | 0.15323 | 3.82 | 0.0001 |
| **ageage_bl** | -0.00376 | 0.00151 | -2.49 | 0.0129 |
| **edu1** | 0.25404 | 0.75267 | 0.34 | 0.7357 |
| **edu2** | 0.35217 | 0.47202 | 0.75 | 0.4556 |
| **edu3** | -0.32147 | 0.52991 | -0.61 | 0.5441 |
| **mastat1** | 0.32669 | 0.71429 | 0.46 | 0.6474 |
| **mastat2** | 0.49219 | 0.46224 | 1.06 | 0.2870 |
| **eversmok** | -0.40862 | 0.35436 | -1.15 | 0.2489 |
| **cigday_prebl0** | -2.07386 | 0.72463 | -2.86 | 0.0042 |
| **cigday_prebl1** | -1.95793 | 1.22059 | -1.60 | 0.1087 |
| **cigday_prebl2** | -0.76630 | 0.95943 | -0.80 | 0.4245 |
| **cigday_prebl3** | -1.87140 | 0.70176 | -2.67 | 0.0077 |
| **drinksday_prebl1** | 1.05483 | 0.97750 | 1.08 | 0.2806 |
| **drinksday_prebl2** | 0.37403 | 0.91705 | 0.41 | 0.6834 |
| **drinksday_prebl3** | -0.20089 | 0.92791 | -0.22 | 0.8286 |
| **bmi_prebl** | -0.26806 | 0.07993 | -3.35 | 0.0008 |
| **dm_prebl** | 2.84795 | 1.32237 | 2.15 | 0.0313 |
| **sbp_prebl** | 0.26825 | 0.01355 | 19.80 | <.0001 |
| **ldlf_prebl** | -0.00518 | 0.00637 | -0.81 | 0.4163 |
| **antichol_prebl** | -1.62034 | 2.03125 | -0.80 | 0.4251 |
| **bpmed_prebl** | 1.23295 | 0.49276 | 2.50 | 0.0124 |
| **exam_1** | 0 | . | . | . |
| **exam_2** | -0.50605 | 0.38896 | -1.30 | 0.1933 |
| **exam_3** | 1.66771 | 0.38263 | 4.36 | <.0001 |
| **cigday_l1** | -0.07314 | 0.03068 | -2.38 | 0.0172 |
| **drinksday_l1_1** | 2.54589 | 1.24695 | 2.04 | 0.0412 |
| **drinksday_l1_2** | 1.68131 | 1.18724 | 1.42 | 0.1568 |
| **drinksday_l1_3** | 1.77221 | 1.19666 | 1.48 | 0.1387 |
| **bmi_l1** | -0.74993 | 0.09544 | -7.86 | <.0001 |
| **dm** | 2.31396 | 1.30055 | 1.78 | 0.0752 |
| **tsdm_inter** | -0.83506 | 0.60816 | -1.37 | 0.1698 |
| **sbp_l1** | 0.48758 | 0.01165 | 41.84 | <.0001 |
| **ldlf_l1** | 0.01711 | 0.00635 | 2.70 | 0.0070 |
| **antichol_l1** | -1.78596 | 0.73812 | -2.42 | 0.0156 |
| **cigday** | 0.00624 | 0.03176 | 0.20 | 0.8442 |
| **drinksday_1** | -4.48954 | 1.25720 | -3.57 | 0.0004 |
| **drinksday_2** | -3.35505 | 1.20513 | -2.78 | 0.0054 |
| **drinksday_3** | -2.78358 | 1.22355 | -2.28 | 0.0229 |
| **bmi** | 1.11908 | 0.08154 | 13.72 | <.0001 |

1. Linear model to estimate LDL-cholesterol

| **Variable** | **Parameter Estimate** | **Standard Error** | **t Value** | **Pr > \|t\|** |
| --- | --- | --- | --- | --- |
| **Intercept** | 17.20912 | 7.78057 | 2.21 | 0.0270 |
| **sex** | 0.54765 | 0.59352 | 0.92 | 0.3562 |
| **age_bl** | 0.52609 | 0.27295 | 1.93 | 0.0540 |
| **ageage_bl** | -0.00676 | 0.00269 | -2.51 | 0.0121 |
| **edu1** | -1.75576 | 1.33942 | -1.31 | 0.1900 |
| **edu2** | -0.10802 | 0.84001 | -0.13 | 0.8977 |
| **edu3** | 1.05332 | 0.94302 | 1.12 | 0.2640 |
| **mastat1** | 0.82842 | 1.27113 | 0.65 | 0.5146 |
| **mastat2** | 0.13557 | 0.82264 | 0.16 | 0.8691 |
| **eversmok** | 0.69843 | 0.63066 | 1.11 | 0.2681 |
| **cigday_prebl0** | 1.33436 | 1.29024 | 1.03 | 0.3011 |
| **cigday_prebl1** | 0.57673 | 2.17247 | 0.27 | 0.7907 |
| **cigday_prebl2** | -2.11506 | 1.70743 | -1.24 | 0.2155 |
| **cigday_prebl3** | 0.40664 | 1.24942 | 0.33 | 0.7448 |
| **drinksday_prebl1** | -1.56742 | 1.73964 | -0.90 | 0.3676 |
| **drinksday_prebl2** | -1.16625 | 1.63195 | -0.71 | 0.4749 |
| **drinksday_prebl3** | -1.62643 | 1.65127 | -0.98 | 0.3247 |
| **bmi_prebl** | -0.51819 | 0.14235 | -3.64 | 0.0003 |
| **dm_prebl** | 5.63114 | 2.35396 | 2.39 | 0.0168 |
| **sbp_prebl** | -0.08768 | 0.02475 | -3.54 | 0.0004 |
| **ldlf_prebl** | 0.25536 | 0.01134 | 22.51 | <.0001 |
| **antichol_prebl** | 5.04499 | 3.61484 | 1.40 | 0.1629 |
| **bpmed_prebl** | -2.78630 | 0.87727 | -3.18 | 0.0015 |
| **exam_1** | 0 | . | . | . |
| **exam_2** | 2.06956 | 0.69226 | 2.99 | 0.0028 |
| **exam_3** | 5.30616 | 0.68180 | 7.78 | <.0001 |
| **cigday_l1** | -0.06224 | 0.05463 | -1.14 | 0.2545 |
| **drinksday_l1_1** | -0.04616 | 2.21964 | -0.02 | 0.9834 |
| **drinksday_l1_2** | -0.37424 | 2.11303 | -0.18 | 0.8594 |
| **drinksday_l1_3** | -0.12818 | 2.12982 | -0.06 | 0.9520 |
| **bmi_l1** | -0.82371 | 0.17056 | -4.83 | <.0001 |
| **dm** | -6.35207 | 2.31488 | -2.74 | 0.0061 |
| **tsdm_inter** | 0.69338 | 1.08239 | 0.64 | 0.5218 |
| **sbp_l1** | 0.03104 | 0.02312 | 1.34 | 0.1794 |
| **ldlf_l1** | 0.51179 | 0.01130 | 45.29 | <.0001 |
| **antichol_l1** | -12.76237 | 1.31405 | -9.71 | <.0001 |
| **cigday** | 0.12249 | 0.05652 | 2.17 | 0.0303 |
| **drinksday_1** | 1.92003 | 2.23922 | 0.86 | 0.3912 |
| **drinksday_2** | 1.63669 | 2.14572 | 0.76 | 0.4456 |
| **drinksday_3** | 0.50219 | 2.17814 | 0.23 | 0.8177 |
| **bmi** | 1.17115 | 0.14699 | 7.97 | <.0001 |
| **sbp** | 0.04659 | 0.02095 | 2.22 | 0.0262 |

1. Logistic model to estimate the probability of taking lipid lowering medication

| **Parameter** | **Estimate** | **Standard Error** | **Wald Chi-Square** | **Pr > ChiSq** |
| --- | --- | --- | --- | --- |
| **Intercept** | -13.3907 | 2.2105 | 36.6964 | <.0001 |
| **sex** | -0.3025 | 0.1353 | 5.0008 | 0.0253 |
| **age_bl** | 0.2660 | 0.0784 | 11.5114 | 0.0007 |
| **ageage_bl** | -0.00250 | 0.000745 | 11.2604 | 0.0008 |
| **edu1** | 0.3666 | 0.2792 | 1.7242 | 0.1891 |
| **edu2** | 0.000536 | 0.1986 | 0.0000 | 0.9978 |
| **edu3** | 0.1041 | 0.2244 | 0.2152 | 0.6427 |
| **mastat1** | 0.1683 | 0.3192 | 0.2779 | 0.5981 |
| **mastat2** | 0.0791 | 0.1880 | 0.1769 | 0.6740 |
| **eversmok** | 0.1630 | 0.1431 | 1.2973 | 0.2547 |
| **cigday_prebl0** | 0.4682 | 0.2807 | 2.7820 | 0.0953 |
| **cigday_prebl1** | 0.7148 | 0.5037 | 2.0133 | 0.1559 |
| **cigday_prebl2** | 0.5934 | 0.3561 | 2.7769 | 0.0956 |
| **cigday_prebl3** | 0.2423 | 0.3019 | 0.6442 | 0.4222 |
| **drinksday_prebl1** | -0.2361 | 0.3456 | 0.4668 | 0.4945 |
| **drinksday_prebl2** | -0.4003 | 0.3198 | 1.5668 | 0.2107 |
| **drinksday_prebl3** | -0.6416 | 0.3283 | 3.8204 | 0.0506 |
| **bmi_prebl** | -0.0701 | 0.0306 | 5.2440 | 0.0220 |
| **dm_prebl** | 0.4220 | 0.4227 | 0.9969 | 0.3181 |
| **sbp_prebl** | 0.00888 | 0.00505 | 3.0953 | 0.0785 |
| **ldlf_prebl** | 0.0239 | 0.00236 | 102.6563 | <.0001 |
| **antichol_prebl** | 1.0266 | 0.6410 | 2.5653 | 0.1092 |
| **bpmed_prebl** | 0.3382 | 0.1701 | 3.9513 | 0.0468 |
| **exam_1** | 0 | . | . | . |
| **exam_2** | -1.2273 | 0.1651 | 55.2602 | <.0001 |
| **exam_3** | -0.3210 | 0.1461 | 4.8310 | 0.0280 |
| **cigday_l1** | -0.0267 | 0.0137 | 3.8224 | 0.0506 |
| **drinksday_l1_1** | 0.2454 | 0.5272 | 0.2167 | 0.6416 |
| **drinksday_l1_2** | 0.3398 | 0.5063 | 0.4503 | 0.5022 |
| **drinksday_l1_3** | 0.3195 | 0.5141 | 0.3863 | 0.5342 |
| **bmi_l1** | -0.0855 | 0.0369 | 5.3815 | 0.0204 |
| **dm** | 0.2963 | 0.4259 | 0.4842 | 0.4865 |
| **tsdm_inter** | -0.0152 | 0.1915 | 0.0063 | 0.9369 |
| **sbp_l1** | 0.00320 | 0.00481 | 0.4417 | 0.5063 |
| **ldlf_l1** | 0.0439 | 0.00260 | 284.8360 | <.0001 |
| **antichol_l1** | 4.9585 | 0.2222 | 497.7919 | <.0001 |
| **cigday** | 0.0212 | 0.0145 | 2.1453 | 0.1430 |
| **drinksday_1** | 0.1282 | 0.4895 | 0.0686 | 0.7933 |
| **drinksday_2** | -0.0554 | 0.4720 | 0.0138 | 0.9065 |
| **drinksday_3** | 0.0801 | 0.4792 | 0.0279 | 0.8673 |
| **bmi** | 0.1499 | 0.0305 | 24.0995 | <.0001 |
| **sbp** | -0.00508 | 0.00441 | 1.3263 | 0.2495 |
| **ldlf** | -0.0532 | 0.00265 | 404.3541 | <.0001 |
