# Supplementary material for: Long-term moderately elevated LDL-cholesterol and blood pressure and risk of coronary heart disease
Source: PLoS One. 2018 Jul 30;13(7):e0200017. doi: 10.1371/journal.pone.0200017 (PMC6066205; doi:10.1371/journal.pone.0200017)
Supplement: S4 Table — (DOCX) [file pone.0200017.s004.docx]

**S4 Table.** **Risk of coronary heart disease (CHD) under different levels, durations and timing of exposure to LDL-cholesterol in the Framingham Offspring Study during 16 years of follow-up time after the 4th examination cycle (1987-1991).**

|  | Duration and exposure to LDL-cholesterol | 16-year risk of CHD^a^ | Population risk ratio^b^ | Population risk difference^b^ | Cumulative percentage intervened on | Average percentage intervened on |
| --- | --- | --- | --- | --- | --- | --- |
| Same exposure during whole study period | 16 years of low LDL (<130 mg/dL)c | 8.2 (7.0 to 9.6) | 1 | 0 | 73 | 38 |
|  | 16 years of moderate LDL (130 to <160 mg/dL) | 8.9 (7.8 to 10.1) | 1.08 (0.95 to 1.22) | 0.69 (-0.41 to 1.68) | 97 | 64 |
|  | 16 years of high LDL (160 to <190 mg/dL) | 9.4 (7.8 to 11.5) | 1.14 (0.92 to 1.46) | 1.16 (-0.75 to 3.46) | 99 | 79 |
|  | 16 years of very high LDL (>190 mg/dL) | 10.3 (7.6 to 13.9) | 1.25 (0.87 to 1.80) | 2.02 (-1.11 to 5.98) | 99 | 91 |
| Moderate LDL in the end of study period | 12 years of low LDL followed by 4 years of moderate LDL | 8.4 (7.3 to 9.6) | 1.02 (0.98 to 1.07) | 0.19 (-0.19 to 0.51) | 96 | 50 |
|  | 8 years of low LDL followed by 8 years of moderate LDL | 8.7 (7.5 to 9.7) | 1.05 (0.97 to 1.12) | 0.44 (-0.26 to 0.93) | 97 | 56 |
|  | 4 years of low LDL followed by 12 years of moderate LDL | 8.7 (7.6 to 9.9) | 1.06 (0.96 to 1.18) | 0.50 (-0.36 to 1.29) | 98 | 62 |
| High LDL in the end of study period | 12 years of low LDL followed by 4 years of high LDL | 8.6 (7.5 to 9.7) | 1.04 (0.97 to 1.14) | 0.35 (-0.30 to 1.04) | 100 | 55 |
|  | 8 years of low LDL followed by 8 years of high LDL | 9.0 (7.7 to 10.3) | 1.09 (0.95 to 1.27) | 0.74 (-0.46 to 2.01) | 100 | 66 |
|  | 4 years of low LDL followed by 12 years of high LDL | 9.3 (7.9 to 10.9) | 1.14 (0.93 to 1.37) | 1.11 (-0.65 to 2.82) | 100 | 76 |
| Very high LDL in the end of study period | 12 years of low LDL followed by 4 years of very high LDL | 8.8 (7.6 to 10.1) | 1.07 (0.95 to 1.25) | 0.58 (-0.42 to 1.89) | 100 | 56 |
|  | 8 years of low LDL followed by 8 years of very high LDL | 9.4 (7.8 to 11.4) | 1.14 (0.92 to 1.47) | 1.15 (-0.73 to 3.48) | 100 | 70 |
|  | 4 years of low LDL followed by 12 years of very high LDL | 10.0 (7.7 to 12.8) | 1.21 (0.89 to 1.66) | 1.75 (-0.97 to 4.89) | 100 | 83 |
| Moderate LDL in the beginning of study period | 4 years of moderate LDL followed by 12 years if low LDL | 8.5 (7.2 to 9.6) | 1.03 (0.99 to 1.06) | 0.27 (-0.13 to 0.49) | 94 | 48 |
|  | 8 years of moderate LDL followed by 8 years of low LDL | 8.6 (7.5 to 9.7) | 1.05 (0.98 to 1.12) | 0.40 (-0.22 to 0.87) | 97 | 55 |
|  | 12 years of moderate LDL followed by 4 years of low LDL | 8.7 (7.7 to 9.9) | 1.06 (0.96 to 1.18) | 0.49 (-0.33 to 1.36) | 97 | 60 |
| High LDL in the beginning of study period | 4 years of high LDL followed by 12 years of low LDL | 8.6 (7.5 to 9.7) | 1.05 (0.98 to 1.12) | 0.40 (-0.20 to 0.91) | 99 | 58 |
|  | 8 years of high LDL followed by 8 years of low LDL | 8.9 (7.8 to 10.2) | 1.08 (0.96 to 1.23) | 0.70 (-0.33 to 1.74) | 100 | 68 |
|  | 12 years of high LDL followed by 4 years of low LDL | 9.1 (7.9 to 10.8) | 1.11 (0.94 to 1.36) | 0.90 (-0.54 to 2.67) | 100 | 76 |
| Very high LDL in the beginning of study period | 4 years of very high LDL followed by 12 years of low LDL | 8.8 (7.7 to 10.0) | 1.07 (0.97 to 1.19) | 0.58 (-0.24 to 1.47) | 100 | 66 |
|  | 8 years of very high LDL followed by 8 years of low LDL | 9.3 (7.9 to 10.9) | 1.13 (0.95 to 1.39) | 1.08 (-0.47 to 2.90) | 100 | 79 |
|  | 12 years of very high LDL followed by 4 years of low LDL | 9.8 (7.9 to 12.5) | 1.19 (0.91 to 1.60) | 1.56 (-0.78 to 4.52) | 100 | 89 |

^a^ There were 218 cases of CHD among 2,972 cohort participants after 39,884 person-years of follow-up. The observed risk was 8.5%.

^b^ In addition to LDL-cholesterol levels, we modeled 7 other covariates in the analysis: examination cycle, cigarette smoking (current smoker, and number of cigarettes per day if smoker), alcohol consumption (standard drinks per day), body mass index, diabetes, systolic blood pressure, and blood pressure medication. All models included lagged values of time-varying covariates plus baseline non-time-varying variables: sex, age, education level, marital status at examination cycle 4, and smoking history at examination cycle 3 of the Framingham Offspring Study.

^c^ Reference category
