# Supplementary material for: Long-term moderately elevated LDL-cholesterol and blood pressure and risk of coronary heart disease
Source: PLoS One. 2018 Jul 30;13(7):e0200017. doi: 10.1371/journal.pone.0200017 (PMC6066205; doi:10.1371/journal.pone.0200017)
Supplement: S5 Table — (DOCX) [file pone.0200017.s005.docx]

**S5 Table.** **Risk of coronary heart disease (CHD) under different levels, durations and timing of exposure to systolic blood pressure (SBP) in the Framingham Offspring Study during 16 years of follow-up time after the 4th examination cycle (1987-1991).**

|  |  | 16-year risk of CHD^a^ | Population risk ratio^b^ | Population risk difference^b^ | Cumulative percentage intervened on | Average percentage intervened on |
| --- | --- | --- | --- | --- | --- | --- |
| Same exposure during whole study period | 16 years of low SBP (<120 mmHg)^c^ | 6.7 (5.5 to 8.1) | 1 | 0 | 85 | 55 |
|  | 16 years of prehypertension (120 to <140 mmHg) | 8.4 (7.3 to 9.5) | 1.26 (1.11 to 1.41) | 1.73 (0.85 to 2.45) | 95 | 55 |
|  | 16 years of stage 1 hypertension (140 to <160 mmHg) | 10.4 (8.9 to 12.1) | 1.55 (1.23 to 1.95) | 3.70 (1.77 to 5.56) | 98 | 73 |
|  | 16 years of stage 2 hypertension (>180 mmHg) | 13.7 (10.5 to 17.0) | 2.04 (1.40 to 2.90) | 6.97 (3.07 to 10.91) | 99 | 89 |
| Prehypertension in the end of study period | 12 years of low SBP followed by 4 years of prehypertention | 7.0 (5.9 to 8.4) | 1.05 (1.02 to 1.10) | 0.36 (0.16 to 0.60) | 96 | 56 |
|  | 8 years of low SBP followed by 8 years of prehypertension | 7.5 (6.4 to 8.7) | 1.12 (1.05 to 1.20) | 0.80 (0.41 to 1.23) | 96 | 56 |
|  | 4 years of low SBP followed by 12 years of prehypertension | 7.9 (6.8 to 9.1) | 1.18 (1.08 to 1.31) | 1.19 (0.60 to 1.80) | 97 | 55 |
| Stage 1 hypertension in the end of study period | 12 years of low SBP followed by 4 years of stage 1 hypertension | 7.6 (6.5 to 8.8) | 1.14 (1.06 to 1.25) | 0.93 (0.42 to 1.50) | 99 | 65 |
|  | 8 years of low SBP followed by 8 years of stage 1 hypertension | 8.6 (7.5 to 9.7) | 1.28 (1.12 to 1.49) | 1.90 (0.88 to 2.92) | 99 | 68 |
|  | 4 years of low SBP followed by 12 years of stage 1 hypertension | 9.6 (8.3 to 10.9) | 1.43 (1.18 to 1.74) | 2.87 (1.33 to 4.30) | 100 | 72 |
| Stage 2 hypertension in the end of study period | 12 years of low SBP followed by 4 years of stage 2 hypertension | 8.6 (7.5 to 10.0) | 1.29 (1.11 to 1.57) | 1.90 (0.82 to 3.29) | 100 | 67 |
|  | 8 years of low SBP followed by 8 years of stage 2 hypertension | 10.5 (8.7 to 12.5) | 1.57 (1.21 to 2.03) | 3.79 (1.66 to 6.05) | 100 | 75 |
|  | 4 years of low SBP followed by 12 years of stage 2 hypertension | 12.1 (9.7 to 15.0) | 1.81 (1.31 to 2.49) | 5.41 (2.40 to 8.67) | 100 | 83 |
| Prehypertension in the beginning of study period | 4 years of prehypertension followed by 12 years of low SBP | 7.1 (5.9 to 8.4) | 1.06 (1.02 to 1.10) | 0.40 (0.17 to 0.61) | 97 | 61 |
|  | 8 years of prehypertension followed by 8 years of low SBP | 7.5 (6.4 to 8.7) | 1.13 (1.05 to 1.20) | 0.85 (0.40 to 1.19) | 98 | 61 |
|  | 12 years of prehypertension followed by 4 years of low SBP | 8.0 (6.8 to 9.1) | 1.20 (1.08 to 1.30) | 1.33 (0.64 to 1.82) | 97 | 60 |
| Stage 1 hypertension in the beginning of study period | 4 years of stage 1 hypertension followed by 12 years of low SBP | 7.5 (6.4 to 8.8) | 1.13 (1.05 to 1.23) | 0.84 (0.43 to 1.35) | 100 | 71 |
|  | 8 years of stage 1 hypertension followed by 8 years of low SBP | 8.5 (7.4 to 9.6) | 1.27 (1.11 to 1.45) | 1.79 (0.86 to 2.71) | 100 | 75 |
|  | 12 years of stage 1 hypertension followed by 4 years of low SBP | 9.5 (8.2 to 10.8) | 1.42 (1.17 to 1.71) | 2.83 (1.34 to 4.15) | 100 | 76 |
| Stage 2 hypertension in the beginning of study period | 4 years of stage 2 hypertension followed by 12 years of low SBP | 8.3 (7.1 to 9.5) | 1.24 (1.10 to 1.45) | 1.62 (0.75 to 2.68) | 100 | 77 |
|  | 8 years of stage 2 hypertension followed by 8 years of low SBP | 10.0 (8.6 to 11.8) | 1.50 (1.19 to 1.91) | 3.31 (1.49 to 5.34) | 100 | 85 |
|  | 12 years of stage 2 hypertension followed by 4 years of low SBP | 11.9 (9.6 to 14.4) | 1.77 (1.30 to 2.38) | 5.17 (2.28 to 8.03) | 100 | 91 |

^a^ There were 218 cases of CHD among 2,972 cohort participants after 39,884 person-years of follow-up. The observed risk was 8.5%.

^b^ In addition to systolic blood pressure levels, we modeled 7 other covariates in the analysis: examination cycle, cigarette smoking (current smoker, and number of cigarettes per day if smoker), alcohol consumption (standard drinks per day), body mass index, diabetes, systolic blood pressure, and lipid lowering medication. All models included lagged values of time-varying covariates plus baseline non-time-varying variables: sex, age, education level, marital status at examination 4, and smoking history at examination 3 of the Framingham Offspring Study.

^c^ Reference category
