# Supplementary material for: Long-term moderately elevated LDL-cholesterol and blood pressure and risk of coronary heart disease
Source: PLoS One. 2018 Jul 30;13(7):e0200017. doi: 10.1371/journal.pone.0200017 (PMC6066205; doi:10.1371/journal.pone.0200017)
Supplement: S2 Fig — Observed values were used for the 4th examination cycle (baseline) and follow-up included examination cycles 5–7. (DOCX) [file pone.0200017.s007.docx]

**S3 Figure.** Mean difference between observed and simulated values and their 95% confidence intervals by examination cycle for the models used for interventions on systolic blood pressure in the Framingham Offspring Study: (a) number of cigarettes smoked per day, (b) number of alcoholic drinks per day, (c) body mass index (BMI), (d) prevalence of diabetes mellitus, (e) systolic blood pressure, (f) LDL-cholesterol and (g) lipid lowering medication. Observed values were used for the 4th examination cycle (baseline) and follow-up included examination cycles 5-7.

(a)

(b)

(c)

(d)

(e)

(f)

(g)
